# Advancing noninvasive glioma classification with diffusion radiomics: Exploring the impact of signal intensity normalization

**DOI:** 10.1093/noajnl/vdae043

**Published:** 2024-03-22

**Authors:** Martha Foltyn-Dumitru, Marianne Schell, Felix Sahm, Tobias Kessler, Wolfgang Wick, Martin Bendszus, Aditya Rastogi, Gianluca Brugnara, Philipp Vollmuth

**Affiliations:** Department of Neuroradiology, Heidelberg University Hospital, Heidelberg, Germany; Division for Computational Neuroimaging, Department of Neuroradiology, Heidelberg University Hospital, Heidelberg, Germany; Department of Neuroradiology, Heidelberg University Hospital, Heidelberg, Germany; Division for Computational Neuroimaging, Department of Neuroradiology, Heidelberg University Hospital, Heidelberg, Germany; Department of Neuropathology, Heidelberg University Hospital, Heidelberg, Germany; Clinical Cooperation Unit Neuropathology, German Cancer Consortium (DKTK), German Cancer Research Center (DKFZ), Heidelberg, Germany; Department of Neurology and Neurooncology Program, Heidelberg University Hospital, Heidelberg University, Heidelberg, Germany; Clinical Cooperation Unit Neurooncology, German Cancer Research Center (DKFZ), Heidelberg, Germany; Department of Neurology and Neurooncology Program, Heidelberg University Hospital, Heidelberg University, Heidelberg, Germany; Clinical Cooperation Unit Neurooncology, German Cancer Research Center (DKFZ), Heidelberg, Germany; Department of Neuroradiology, Heidelberg University Hospital, Heidelberg, Germany; Department of Neuroradiology, Heidelberg University Hospital, Heidelberg, Germany; Division for Computational Neuroimaging, Department of Neuroradiology, Heidelberg University Hospital, Heidelberg, Germany; Department of Neuroradiology, Heidelberg University Hospital, Heidelberg, Germany; Division for Computational Neuroimaging, Department of Neuroradiology, Heidelberg University Hospital, Heidelberg, Germany; Department of Neuroradiology, Heidelberg University Hospital, Heidelberg, Germany; Division for Computational Neuroimaging, Department of Neuroradiology, Heidelberg University Hospital, Heidelberg, Germany; Division for Computational Radiology & Clinical AI, Department of Neuroradiology, Bonn University Hospital, Bonn, Germany

**Keywords:** diffusion magnetic resonance imaging, genotype, glioma, IDH mutation, magnetic resonance imaging

## Abstract

**Background:**

This study investigates the influence of diffusion-weighted Magnetic Resonance Imaging (DWI-MRI) on radiomic-based prediction of glioma types according to molecular status and assesses the impact of DWI intensity normalization on model generalizability.

**Methods:**

Radiomic features, compliant with image biomarker standardization initiative standards, were extracted from preoperative MRI of 549 patients with diffuse glioma, known IDH, and 1p19q-status. Anatomical sequences (T1, T1c, T2, FLAIR) underwent N4-Bias Field Correction (N4) and WhiteStripe normalization (N4/WS). Apparent diffusion coefficient (ADC) maps were normalized using N4 or N4/z-score. Nine machine-learning algorithms were trained for multiclass prediction of glioma types (IDH-mutant 1p/19q codeleted, IDH-mutant 1p/19q non-codeleted, IDH-wild type). Four approaches were compared: Anatomical, anatomical + ADC naive, anatomical + ADC N4, and anatomical + ADC N4/z-score. The University of California San Francisco (UCSF)-glioma dataset (*n* = 409) was used for external validation.

**Results:**

Naïve-Bayes algorithms yielded overall the best performance on the internal test set. Adding ADC radiomics significantly improved AUC from 0.79 to 0.86 (*P* = .011) for the IDH-wild-type subgroup, but not for the other 2 glioma subgroups (*P* > .05). In the external UCSF dataset, the addition of ADC radiomics yielded a significantly higher AUC for the IDH-wild-type subgroup (*P* ≤ .001): 0.80 (N4/WS anatomical alone), 0.81 (anatomical + ADC naive), 0.81 (anatomical + ADC N4), and 0.88 (anatomical + ADC N4/z-score) as well as for the IDH-mutant 1p/19q non-codeleted subgroup (*P* < .012 each).

**Conclusions:**

ADC radiomics can enhance the performance of conventional MRI-based radiomic models, particularly for IDH-wild-type glioma. The benefit of intensity normalization of ADC maps depends on the type and context of the used data.

## Lay Summary

Gliomas are tumors that originate in the brain. These tumors are classified into 3 main genetic groups: IDH-mutant 1p/19q codeleted, IDH-mutant 1p/19q non-codeleted, IDH-wild type. Knowing which genetic group a glioma belongs to is important for predicting how the tumor will behave. At present, this can only be achieved through genetic testing of tissue obtained during surgery. The authors of this study aimed to determine whether the genetic group of glioma could be predicted using magnetic resonance imaging (MRI). To do this, they developed computer models that combined a special MRI sequence, which detects the movement of water in tumor cells (ADC), with standard anatomic MRI scans. They then tested their models using publicly available data. Their results show that the addition of features from the ADC sequences improved the prediction of some, but not all, genetic types of gliomas.

**Key Points**

1. Radiomics from apparent diffusion coefficient (ADC) maps can improve the performance for classifying glioma molecular types.
2. N4/z-score intensity normalization of ADC maps can lead to better generalizability.

**Importance of the Study**

Accurate tumor characterization is nowadays mandatory for choosing the appropriate glioma therapy. Radiomic feature-based models have recently shown convincing performance in the noninvasive detection of mutations in gliomas, and the addition of functional MRI sequences such as MRI-diffusion has even led to an increase in performance. Intensity normalization of conventional MRI sequences led to greater generalizability of the models, but whether this also applies to functional MRI sequences is still uncertain. Considering the mixed evidence from the literature regarding apparent diffusion coefficient (ADC) normalization, our study suggests a cautious approach. We reevaluated the role of ADC-based radiomics in glioma subtype prediction and explored the potential of intensity normalization of ADC images. However, the evidence does not conclusively support a significant enhancement in generalizability for multi-center datasets. Future research could consider ADC map normalization, yet the clear advantage of this practice is not definitively established.

In 2021, the World Health Organization presented the fifth edition of the classification of tumors of the central nervous system,^1,2^ which classifies adult gliomas into 3 types based on isocitrate dehydrogenase (IDH) and 1p19q status: Astrocytoma, IDH mutant (IDH-mut); Oligodendroglioma, IDH-mutant and 1p/19q codeleted; and Glioblastoma, IDH wild type (IDH-wt). Both machine and deep-learning-based models have shown encouraging results in the noninvasive detection of imaging phenotypes associated with key molecular alterations, through the analysis of radiomic features, which consists of the fully automated extraction of quantitative imaging descriptors of the tumor related to its intensity, shape, volume, and texture.^3–6^ A major disadvantage of models based on radiomic features is the lack of reporting standards, which particularly for MRI-based features hinders the reproducibility of results.^7,8^ Recent work has shown increased generalizability of radiomics-based models through intensity normalization of anatomical MRI sequences.^9^

Furthermore, recent studies have demonstrated how the addition of quantitative data derived from advanced MRI sequences such as diffusion—(DWI) and perfusion-weighted imaging to radiomic-based models allowed an improvement in prediction performance for genetic glioma types.^10–13^ Apparent diffusion coefficient (ADC) maps are generated from DWI and reflect barriers to the Brownian motion of water molecules in each image voxel.^14,15^ To date, investigations have exclusively examined the normalization of ADC values as a strategy to mitigate the disparity observed in reported thresholds pertaining to ADC values within the context of survival correlation in glioblastoma, showing a performance increase through normalization.^16^

In this present study, we explored the advantages of incorporating ADC radiomics alongside conventional sequence-based radiomics models for simultaneous prediction of IDH and 1p19q status in glioma patients. Additionally, we assessed how the intensity normalization of ADC maps influenced the models’ generalizability. For our investigation, we employed a large retrospective dataset from a single institution for model development, training, and internal testing. Furthermore, we conducted external testing using a publicly available dataset.

## Materials and Methods

This retrospective study was approved by the internal ethics committee, and the requirement to obtain informed consent was waived (S-784 2018).

The study included adult patients who had a confirmed primary glioma diagnosis according to the World Health Organization 2021 classification and received a preoperative MRI between March 2009 and July 2020 in the Department of Neuroradiology at Heidelberg University Hospital (Heidelberg, Germany; *n* = 621). For all patients, IDH and 1p/19q status were available based on DNA methylation profiling with the Infinium HumanMethylation450 or HumanMethylationEPIC bead chip.^17^ Based on this information, the gliomas were categorized as IDH-wt, IDH-mut 1p/19q codeleted, and IDH-mut 1p/19q non-codeleted gliomas. Subsequently, *n* = 3 cases were excluded due to insufficient quality of MRI images (eg, motion artifacts), *n* = 72 cases due to lack of diffusion imaging and *n* = 3 cases due to data processing errors. In conclusion, the internal data set (HD) consisted of *n* = 549 patients. The HD dataset was acquired during a routine clinical examination using a 3T MRI machine (Magnetom Verio, Trio TIM, or Skyra, Siemens Healthcare). The imaging protocol included T1-weighted 3D images both before (T1) and after (cT1) administration of a bolus of 0.1 mmol/kg gadoterate meglumine (Dotarem, Guerbet) as well as axial 2D FLAIR and T2-weighted images.^18^ ADC maps were generated directly on the scanner. A detailed description of the MRI acquisition parameters can be found in Supplementary Material.

For the external validation, a publicly available preoperative MRI dataset from the University of California San Francisco (UCSF) with 501 patients was used.^19^ From this dataset, *n* = 91 cases were removed due to unknown 1p/19q status and *n* = 1 due to a broken ADC map, resulting in *n* = 409 patients. Information on the sequences included in the datasets is found at (https://doi.org/10.7937/tcia.bdgf-8v37). A concise overview of the MRI protocol is presented in Supplementary Material. For an exhaustive delineation of the protocol, refer to the comprehensive description provided in the original publication.

### Image Preprocessing and Tumor Segmentation

Images from the HD dataset underwent processing using established and publicly available software, as previously outlined.^20^ In brief, this process encompassed 3 key steps: (I) brain extraction accomplished through the HD-BET tool, which employs neural-network-based techniques,^21^ (II) rigid registration of image volumes to the native T1-w image via FSL (FMRIB, Oxford, UK), and (III) automated deep-learning-based tumor segmentation into distinct components (contrast-enhancing, T2-FLAIR, and necrotic) using a modified version of HD-GLIO [NeuroAI-HD/HD-GLIO]. Subsequently, segmentations underwent visual inspection, with adjustments made by MF (a neuroradiology resident with 5 years of experience) as needed. A manual correction was required in 100 of the 549 cases (18.2%). ADC maps were generated automatically during MRI acquisition by Syngo software (Siemens Healthcare, Erlangen, Germany), and the brain extraction and registration processes were consistent with the previously described methods. The UCSF dataset consisting of preprocessed sequences and a multi-compartment tumor segmentation into contrast-enhancing, T2/FLAIR hyperintense as well as necrotic tumor parts were downloaded via https://doi.org/10.7937/tcia.bdgf-8v37.

As previous work has shown that intensity normalization of anatomical sequences (T1 pre- and post-contrast, T2, FLAIR) can increase the generalizability of radiomics-based prediction models,^9^ the anatomical sequences were normalized by N4 bias field correction followed by WhiteStripe normalization. For the ADC maps 3 normalization approaches were compared: (1) no normalization (naive), (2) N4 bias field correction (N4), and (3) N4/z-score, since the WhiteStripe normalization is not recommended by the developers for ADC maps, we used a Z-score normalization instead. N4 bias field correction, as well as Z-score and WhiteStripe normalization, were performed using the ANTsR and WhiteStripe packages implemented in R (R version 4.0.2., R Foundation for Statistical Computing, https://github.com/muschellij2/WhiteStripe).^22^

**Figure 1.**
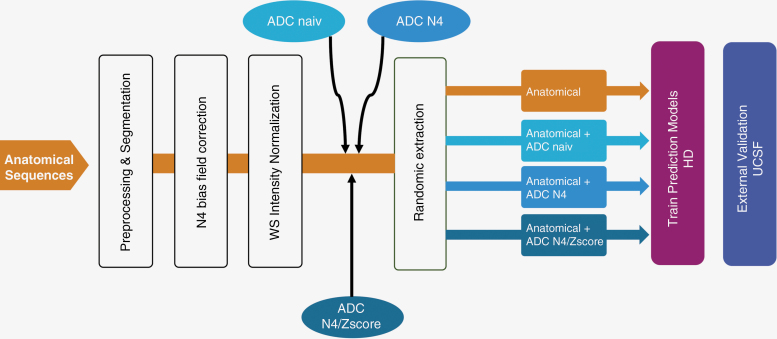
Schematic representation of the study structure. The anatomical sequences were normalized with N4 Bias Field Correction followed by White Stripe normalization (N4/WS). Four different conditions were compared: Anatomical sequences alone (anatomical), anatomical sequences + unnormalized apparent diffusion coefficient (ADC; anatomical + ADC naive), anatomical sequences + ADC with N4 Bias Field Correction (anatomical + ADC N4), or anatomical sequences + ADC N4 Bias Field Correction followed by z-score normalization (anatomical + ADC N4/z-score). Using the training dataset from HD (*n* = 439), 9 different Machine-Learning models were trained for each condition and validated using the holdout test dataset (*n* = 110). External validation was performed using University of California San Francisco data set (*n* = 409).

To assess the influence of diffusion imaging and its intensity normalization on the performance and generalizability of radiomic-based prediction of molecular glioma types, we compared 4 different approaches: (1) radiomics from anatomical sequences alone (N4/WS), (2) anatomical (N4/WS) + ADC naive, (3) anatomical (N4/WS) + ADC N4, and (4) anatomical (N4/WS) + ADC N4/z-score. A graphical representation of the study design is shown in Figure 1.

### Radiomic Feature Extraction

Feature extraction was performed in Python (version 3.8.5) using the open-source Python package PyRadiomics (https://pyradiomics.readthedocs.io).^23^ Features were extracted from the entire tumor. For this purpose, the previously created masks of the different tumor compartments, namely contrast-enhancement, T2/FLAIR hyperintensity, and necrosis were combined into a single mask. Only radiomics that have been described as reproducible by the image biomarker standardization initiative were extracted.^24^ 104 radiomics were extracted per sequence. This included 13 Shape-based, 17 First Order, 23 GLCM (Gray Level Co-occurrence Matrix), 16 GLRLM (Gray Level Run Length Matrix), 16 GLSZM (Gray Level Size Zone Matrix), 14 GLDM (Gray Level Dependence Matrix), and 5 NGTDM (Neighboring Gray Tone Difference Matrix). A list of all extracted radiomics is shown in Supplementary Table 1. As radiomics based on the morphology of the tumor are the same for all 4 sequences, shape features were only kept from the T1 pre-contrast sequence. This resulted in a total number of 377 features for the models without ADC and a total number of 445 radiomics with the addition of the ADC sequence.

### Data Analysis

The HD dataset was divided into a training and a test dataset with a 0.8 ratio while ensuring equal distribution of the 3 subclasses. To avoid overfitting, we performed a feature selection on the training dataset in 2 steps. First, we calculated Pearson correlation between the features and removed 1 of the 2 features that correlated greater than 0.9. In the second step, an ANOVA F-statistic was performed for each feature. Using the empirical rule 5 cases per feature^25^ from the *n* = 439 training data set, we selected the 88 radiomics features with the highest F-statistic for the final models.^26^ These features were also applied to the test and external UCSF validation dataset.

Machine-learning models were built with the scikit-learn and xgboost package in Python (version 3.8.5) for a 3-class classification based on a One-vs-Rest approach. We compared 9 different algorithms, namely logistic regression, linear discriminant analysis, k-nearest neighbor (KNN), decision tree (CART), Naïve-Bayes (NB), C-support vector classification (SVM), random forest, extra-trees classifier (ET) and eXtreme gradient boosting (XGBC).

To balance the training dataset undersampling of the IDH-wt group and SMOTE oversampling of the IDH-mut groups was performed. The desired sample size for each class corresponded to the sample size of the training dataset (*n* = 439) divided by the number of classes (*n* = 3), resulting in a final sample size of *n* = 146 for each of the 3 classes. The statistics of the final training data set after over- and undersampling are shown in Supplementary Table 8.

The performance of the models was evaluated with areas under the receiver operating characteristic curve (AUC), sensitivity, specificity, confusion matrix, and accuracy. Confidence intervals were calculated using bootstrapping. To compare the AUC of the models, we used DeLong’s test. The *P*-values were corrected, for each dataset separately, using false discovery rate. Wilcoxon–Mann–Whitney test was used to compare continuous and Chi-squared test was used to compare categorical data. A *P*-value < .05 was considered significant for all analyses.

## Results

The HD dataset comprised IDH-wt gliomas (*n* = 400), IDH-mutated and 1p/19q non-codeleted (IDH-mut 1p/19q non-codel) gliomas (*n* = 75) as well as IDH-mutated and 1p19q codeleted (IDH-mut 1p/19q codel) gliomas (*n* = 74). The specifications of the included patients from the HD and UCSF datasets are summarized in Table 1.

**Table 1.** Patient Demographic Characteristics. The *P*-value Indicates the Significance Level Between the Two Datasets

Parameter	Data	All Classes	IDH mut + 1p/19q codeletion	IDH mut + 1p/19q non-codeletion	IDH wt
Total no. of patients	HD	549	74	75	400
	UCSF	409	15	84	310
Female (*n*[%])	HD	240 (44)	35 (47)	26 (35)	179 (45)
	UCSF	171 (42)	5 (33)	34 (40)	132 (43)
	*P*-value	0.87	0.87	0.87	0.87
Mean age (y)	HD	57 ± 15	47 ± 14	40 ± 12	63 ± 12
	UCSF	56 ± 15	44 ± 14	38 ± 11	61 ± 12
	*P*-value	0.57	0.87	0.6	0.87

**Figure 2.**
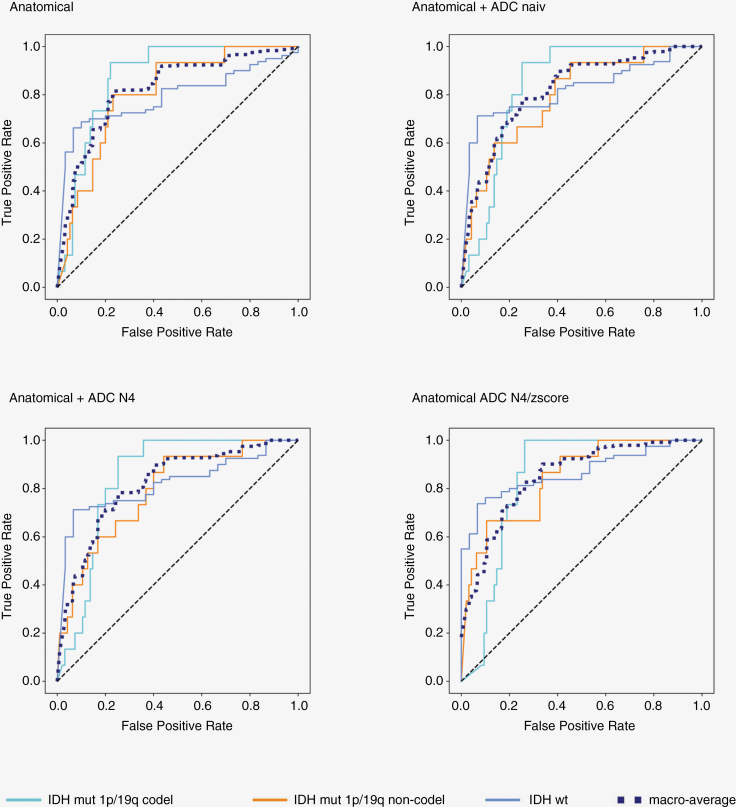
Naïve-Bayes model performance on holdout test dataset (*n* = 110) of HD to classify IDH-wt versus IDH-mut 1p/19q codel versus IDH-mut 1p/19q non-codel via radiomic features across 4 different conditions: Anatomical sequences alone (anatomical), anatomical sequences + unnormalized apparent diffusion coefficient (ADC; anatomical + ADC naive), anatomical sequences + ADC with N4 Bias Field Correction (anatomical + ADC N4) or anatomical sequences + ADC N4 Bias Field Correction followed by z-score normalization (anatomical + ADC N4/z-score). The anatomical sequences were normalized with N4 Bias Field Correction followed by White Stripe normalization.

Among the 9 different tested machine-learning algorithms the NB algorithm showed the most balanced ratio of sensitivity and specificity and simultaneously a good AUC for all different approaches and subclasses (Supplementary Tables 2 and 3). NB reached a macro-average AUC for the multi-class detection of molecular characteristics of 0.82 (95% confidence interval^12^ 0.75–0.88) using anatomical N4/WS data. The scores remained consistent including radiomics from ADC naive or ADC N4 (AUC 0.82; 95% CI: 0.73–88 and 95% CI: 0.73–0.87, respectively). The highest score (AUC = 0.85; 95% CI: 0.77–0.90) was observed using anatomical N4/WS + ADC N4/z-score in the HD test set. For the IDH-wt class, detection rates varied, ranging from AUC 0.79 (95% CI: 0.68–0.86) using anatomical N4/WS alone to AUC 0.85 (95% CI: 0.80–0.92) using anatomical N4/WS + ADC N4/z-score. The addition of ADC improved IDH-wt class prediction significantly (FDR-adjusted *P* = .011), regardless of whether ADC has been normalized or not. There was no significant difference between the addition of naïve ADC radiomics and ADC N4 normalized (FDR-adjusted *P* = .53) or ADC N4 z-score normalized (FDR-adjusted *P* = .07) radiomics. For IDH-mutant 1p/19q non-codeleted and IDH-mutant 1p/19q codeleted groups, the AUC scores showed only little variation across different data combinations (Table 2) without significant difference in model performance (FDR-adjusted *P* ≥ .14 each). Figure 2 shows all corresponding ROC curves. The ROC curves obtained with the 8 other tested models are shown in Supplementary Figures 1 and 2. The respective accuracy values including the 95% CI are shown in Supplementary Tables 2 and 3.

**Table 2.** Naïve-Bayes Model Performance on Holdout Test Dataset (*n* = 110) of HD and External University of California San Francisco (*n* = 409) dataset

Model	Parameter	IDH wt	IDH mut + 1p/19q non-codeletion	IDH mut + 1p/19q codeletion	Macro-average
*Test set HD*					
Anatomical N4/WS	AUC	0.79	0.81	0.87	0.82
	95% CI	0.68–0.86	0.65–0.89	0.79–0.93	0.75–0.88
Anatomical N4/WS + ADC naive	AUC	0.82	0.80	0.84	0.82
	95% CI	0.70–0.88	0.63–0.89	0.76–0.91	0.73–0.88
Anatomical N4/WS + ADC N4	AUC	0.82	0.79	0.84	0.82
	95% CI	0.73–0.87	0.60–0.88	0.75–0.90	0.73–0.87
Anatomical N4/WS + ADC N4/z-score	AUC	0.85	0.84	0.84	0.85
	95% CI	0.80–0.92	0.72–0.93	0.75–0.90	0.77–0.90
*UCSF*					
Anatomical N4/WS	AUC	0.80	0.80	0.81	0.80
	95% CI	0.71–0.82	0.74–0.85	0.61–0.93	0.70–0.84
Anatomical N4/WS + ADC naive	AUC	0.81	0.79	0.81	0.81
	95% CI	0.73–0.83	0.73–0.84	0.60–0.92	0.72–0.84
Anatomical N4/WS + ADC N4	AUC	0.81	0.79	0.80	0.80
	95% CI	0.73–0.83	0.73–0.83	0.62–0.91	0.70–0.84
Anatomical N4/WS + ADC N4/z-score	AUC	0.88	0.86	0.83	0.85
	95% CI	0.83–0.91	0.80–0.89	0.60–0.93	0.78–0.90

**Figure 3.**
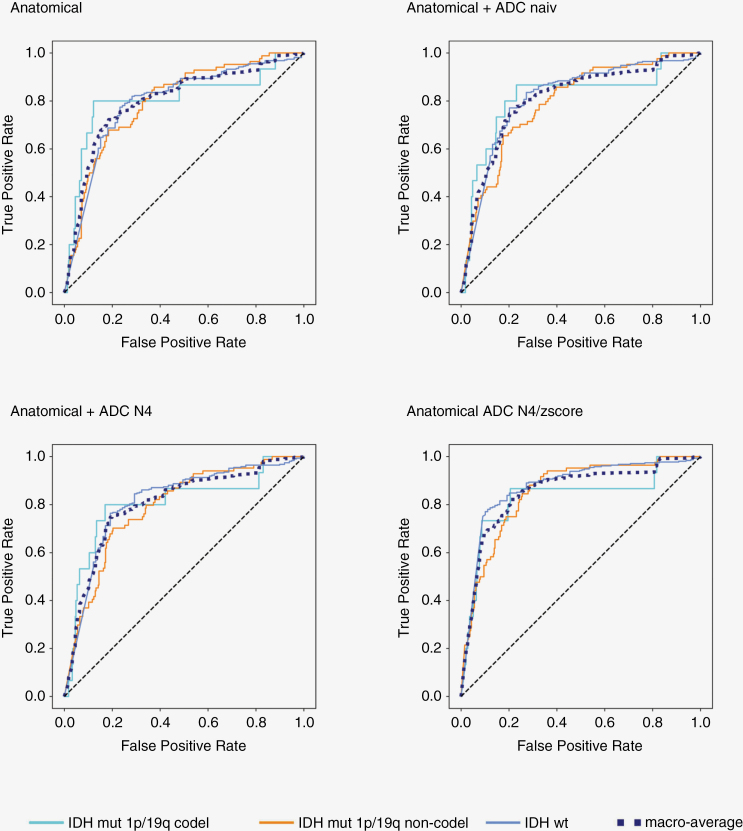
ROC curves of Naïve-Bayes model of external validation with University of California San Francisco data set to differentiate between IDH-wt versus IDH-mut 1p/19q codel versus IDH-mut 1p/19q non-codel across different conditions: anatomical sequences alone (anatomical), anatomical sequences + unnormalized apparent diffusion coefficient (ADC; anatomical + ADC naive), anatomical sequences + ADC with N4 Bias Field Correction (anatomical + ADC N4) or anatomical sequences + ADC N4 Bias Field Correction followed by z-score normalization (anatomical + ADC N4/z-score). The anatomical sequences were normalized with N4 Bias Field Correction followed by White Stripe normalization.

Applying the NB model to the external UCSF dataset, we noticed a similar trend in macro-average AUC scores, with the highest score (AUC 0.85; 95% CI: 0.78–0.90) for the anatomical N4/WS + ADC N4/z-score combination. Individual detection rates for molecular mutation classes showed improved performance through N4/z-score normalization of ADC for the IDH-wt and IDH-mutant 1p/19q non-codeleted classes. In detail, in the IDH-wt class, the combination of anatomical N4/WS + ADC N4/z-score with AUC 0.88; 95% CI: 0.83–0.91 showed the best performance, compared to anatomical N4/WS alone AUC 0.80; 95% CI: 0.71–0.82 (FDR adjusted *P* < .0001) as well as compared to anatomical N4/WS + ADC naïve or anatomical N4/WS + ADC N4 with AUC 0.81; 95% CI: 0.73–0.83 (FDR adjusted *P* = .001) each. In the IDH-mutant 1p/19q non-codeleted class, the combination of anatomical N4/WS + ADC N4/z-score with AUC 0.86; 95% CI: 0.80–0.89 outperformed the other approaches of anatomical N4/WS alone AUC 0.80; 95% CI: 0.74–0.85 (FDR adjusted *P* = .012) as well as anatomical N4/WS + ADC naïve AUC 0.79; 95% CI: 0.73–0.84 (FDR adjusted *P* = .006) or anatomical N4/WS + ADC N4 with AUC 0.79; 95% CI: 0.73–0.83 (FDR adjusted *P* = .01). For the IDH-mutant 1p/19q codeleted class, detection rates varied slightly, ranging from AUC 0.80 (95% CI: 0.70–0.84) using anatomical N4/WS alone to AUC 0.85 (95% CI: 0.78–0.90) using anatomical N4/WS + ADC N4/z-score, without any significant difference in model performance (FDR adjusted *P* ≥ .61 each). Figure 3 shows all corresponding ROC curves. All corrected *P*-values of the individual classes for the HD test and UCSF dataset as well as the confusion matrix are shown in Supplementary Tables 4–7.

## Discussion

Prior research has revealed enhanced performance in glioma-type prediction by incorporating ADC radiomics and normalizing conventional MRI sequences, but the impact of ADC map intensity normalization remains unexplored. In this study, we aimed to reevaluate the described benefit of ADC radiomics for the prediction of glioma types and to investigate the relevance of intensity normalization of ADC maps as well as its impact on the predictive power of machine-learning algorithms. In our study, the benefit of incorporating ADC radiomics into the analysis was not uniformly observed across all glioma types. The addition of ADC data significantly improved the prediction of IDH-wt status, irrespective of whether the ADC was normalized. In contrast, there was no performance improvement in the IDH-mut classes. Applying the developed NB models to external UCSF data, we observed performance improvements with the inclusion of N4/z-score normalized ADC data only for IDH-wt and IDH-mut 1p/19q non-codel classes, where anatomical N4/WS + ADC N4/z-score outperformed other approaches. Notably, the model’s performance for the IDH-mut 1p/19q codel class remained stable across different approaches. Our results confirm the added value of advanced MRI with DWI over anatomical MRI sequences alone for multiclass prediction of molecular glioma types. Moreover, we demonstrated that while the model’s performance remained unaffected by ADC map intensity normalization in internal testing, its performance can become contingent on intensity normalization when deploying the models on external data.

The addition of ADC radiomics led to a performance increase for the prediction of the IDH-wt group in the internal test dataset, regardless of whether the ADC maps were normalized or not. However, when examining the external UCSF dataset, the addition of N4/z-score normalized radiomics for ADC outperformed the other approaches for both the IDH-wt and IDH-mut non-codel groups. These results are in part consistent with the recently published work of Guo et al.,^11^ which showed a significant performance increase for prediction of IDH-wt and IDH-mut non-codel group in both the internal and external dataset by adding normalization-naïve ADC radiomics using a random forest multiclass model. The fact that in the external validation, in contrast to Guo et al., we only saw a performance increase with normalized, but not naïve ADC radiomics, highlights the importance of intensity normalization of ADC for improving the generalizability of radiomic-based prediction models. ADC, as an imaging biomarker, reflects the underlying histopathology of gliomas. It highlights the relationship between cellular density and water diffusion in gliomas, with aggressive tumors showing lower ADC values due to high cellular density restricting water diffusion, and less aggressive tumors exhibiting higher ADC values.^27^ Our study confirms that ADC radiomics significantly improves IDH-wt and IDH-mut non-codel glioma prediction, a finding consistent with previous research indicating that IDH-mut gliomas show higher ADC values compared to IDH wt^28^ and texture features of ADC are useful for determining the 1p/19q status.^28–30^

Unlike anatomical MRI sequences, functional sequences are presented in absolute rather than arbitrary values; however, due to varying ADC cutoffs associated with survival, some researchers have suggested utilizing normalized ADC values instead of absolutes,^16^ although it is important to note that these studies primarily focus on statistical values rather than radiomics derived from ADC maps. Schmeel discussed in 2018 in an editorial the challenges of variability in quantitative DWI across different scanners and sites. He emphasizes the difficulty in achieving consistent ADC measurements, which is crucial in oncologic imaging. The editorial suggests that focusing on ADC change and normalized ADC values, rather than absolute measurements, could aid multi-center studies. Furthermore, it also highlights the need for standardization in DWI protocols and the potential benefits of comparative analysis and normalization techniques in overcoming measurement biases in ADC values.^31^ Only a few papers have investigated the repeatability of radiomic features from ADC maps and these show contradictory results, leaving mixed reports uncertain about whether normalization of the ADC might be required or not to overcome variation between MRI systems.^32,33,31^ Consistent with existing literature, our findings present inconclusive evidence on the impact of ADC normalization. The normalization showed no significant benefit in our internal test dataset. However, it demonstrated utility in enhancing the prediction performance for IDH-wt and IDH-mut 1p/19q non-codeleted classes but not IDH-mut 1p/19q codeleted classes in the external validation dataset.

Our study has some limitations. First, in our study, molecular testing for EGFR or TERT mutations in IDH-wt patients was not conducted. Consequently, the IDH-wt class encompasses both IDH-wt glioblastomas and IDH-wt gliomas of histological grade 2 or 3, not otherwise specified. However, thereby we have ensured that our predictions are not influenced by tumor grade, focusing instead solely on IDH and 1p/19q status. Second, to avoid any bias when evaluating the model performance of various normalization approaches, we did not perform hyperparameter tuning, such as grid search, which may allow us to increase the model performance. Instead, we maintained the model parameters by default. Third, we did not examine novel normalization methods like those based on Convolutional Neural Networks,^34^ but limited ourselves to z-score and WhiteStripe normalization, which are quite similar methods. However, this study provides a good base, since these 2 methods are among the most widely used in radiomic research.^35^ The comparison to novel approaches needs to be explored in future works. Fourth, we have not investigated the physiological explainability and biological interpretability of the ADC-based radiomics. Future research may be able to elucidate the biological significance of the radiomic model using multi-omics data, such as transcriptome or protein sequencing data.

In summary, in this study, we were able to reevaluate the importance of ADC-based radiomics to glioma class prediction. Furthermore, we highlighted a nuanced advantage in the application of intensity normalization to ADC images within radiomic-based predictive models, particularly when applied in diverse external settings. This finding suggests a specific, context-dependent utility of intensity normalization in enhancing the predictive accuracy of radiomic models in heterogeneous datasets.

## Supplementary Material

vdae043_suppl_Supplementary_Material
