# Supplementary material for: Advancing noninvasive glioma classification with diffusion radiomics: Exploring the impact of signal intensity normalization
Source: Neurooncol Adv. 2024 Mar 22;6(1):vdae043. doi: 10.1093/noajnl/vdae043 (PMC11003539; doi:10.1093/noajnl/vdae043)

Supplementary Figure 1

A (Logistic Regression)

Anatomical

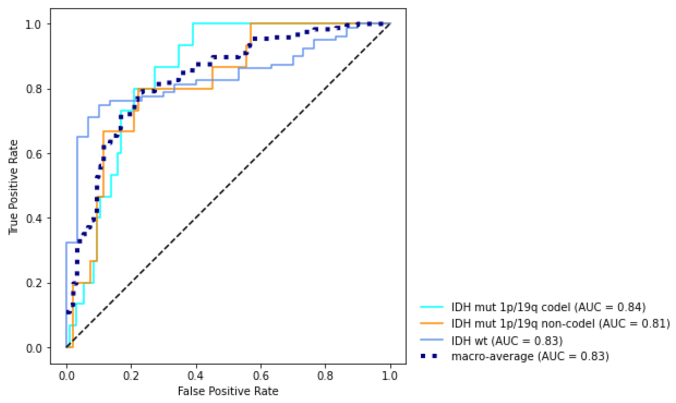

Anatomical + ADC naiv

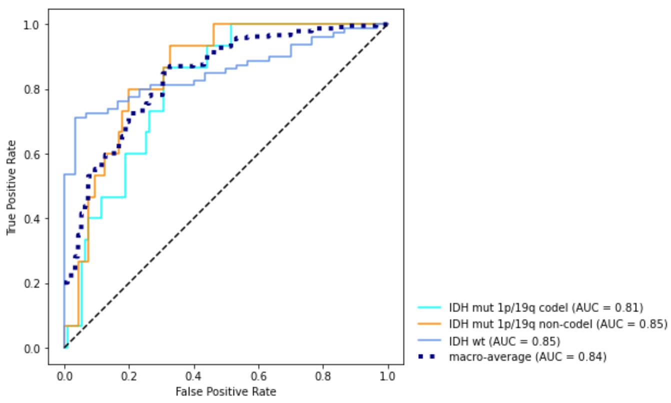

Anatomical + ADC N4

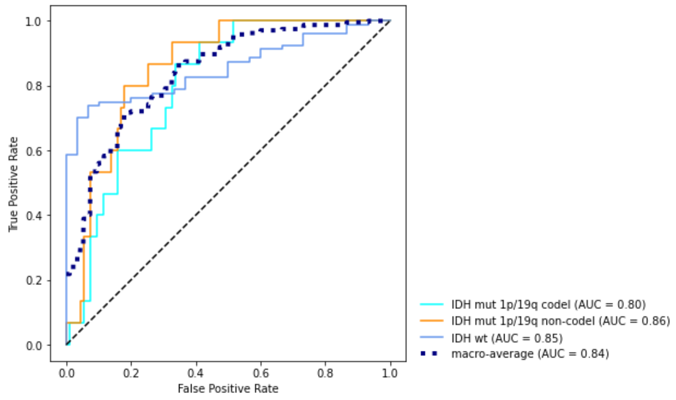

Anatomical + ADC N4/zscore

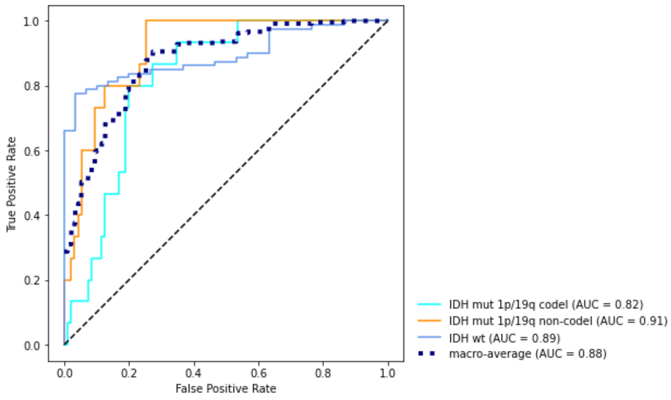

B (Linear Discriminant Analysis)

Anatomical

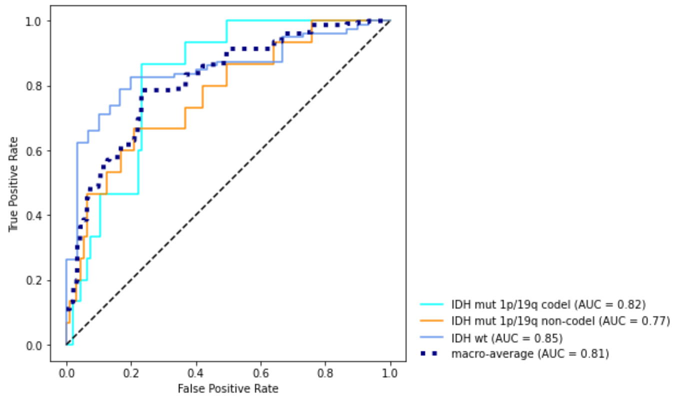

Anatomical + ADC naiv

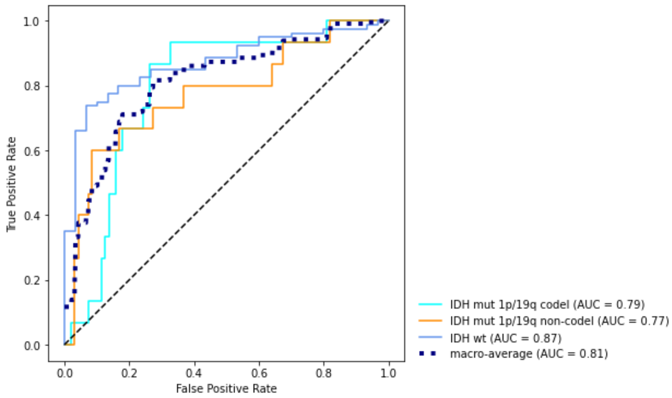

Anatomical + ADC N4

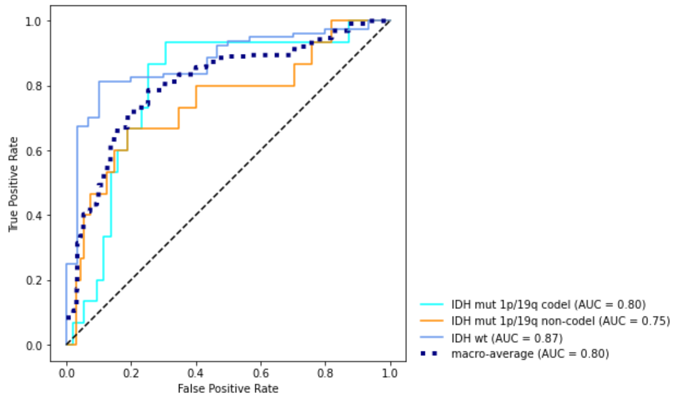

Anatomical + ADC N4/zscore

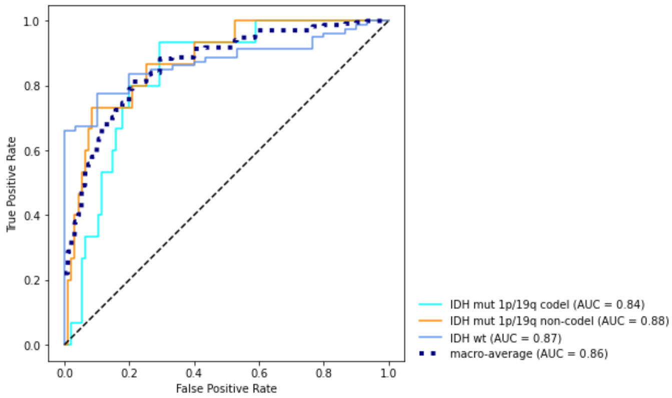

C (k-nearest neighbor)

Anatomical

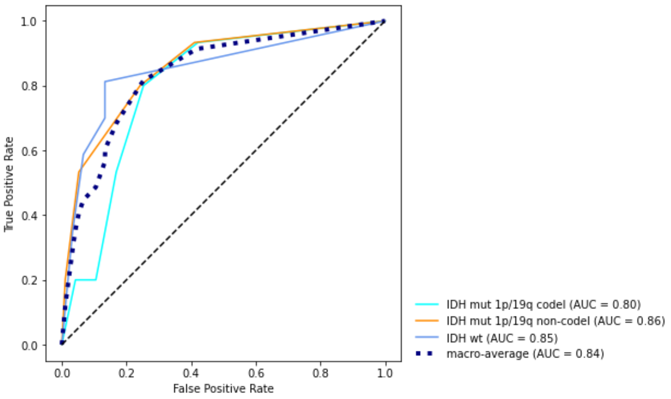

Anatomical + ADC naiv

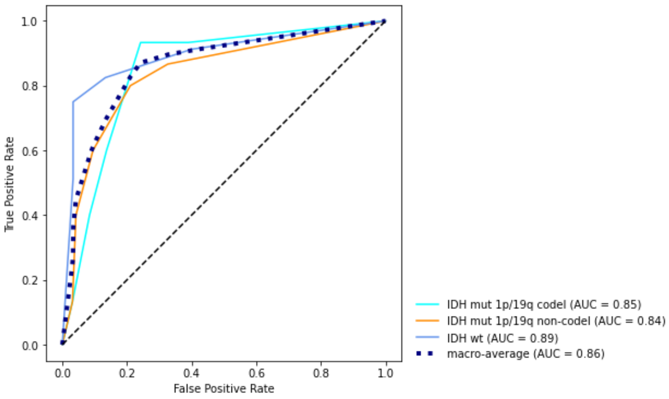

Anatomical + ADC N4

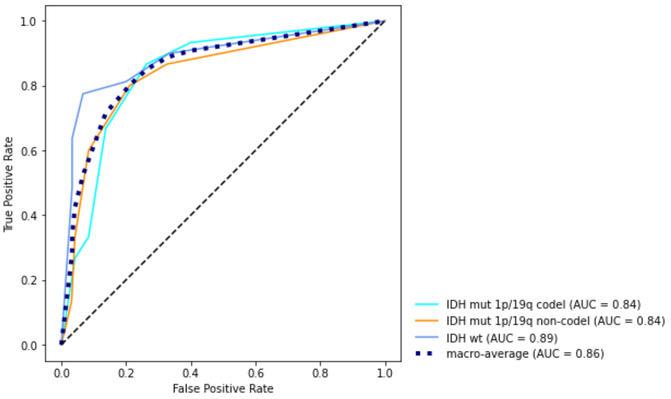

Anatomical + ADC N4/zscore

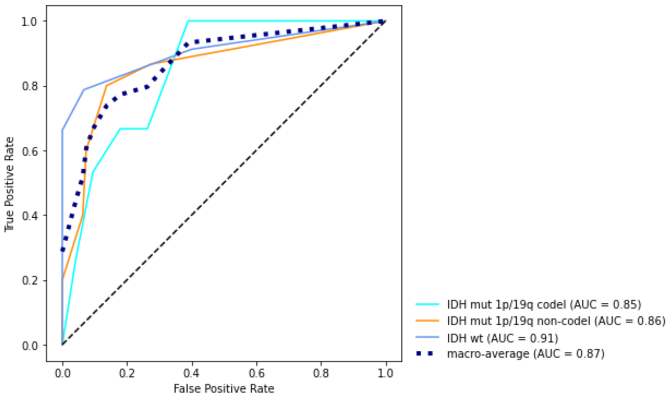

D (Decision tree)

Anatomical

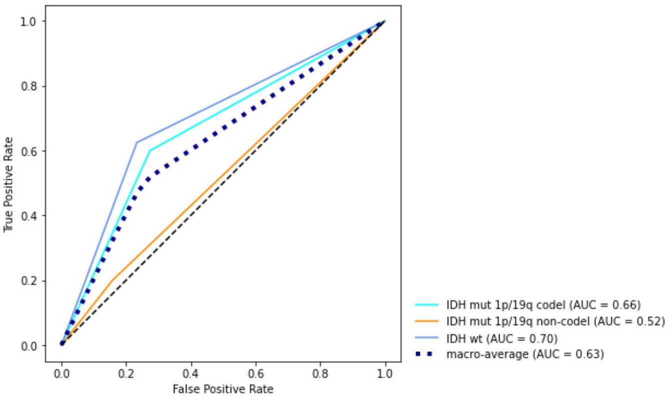

Anatomical + ADC naiv

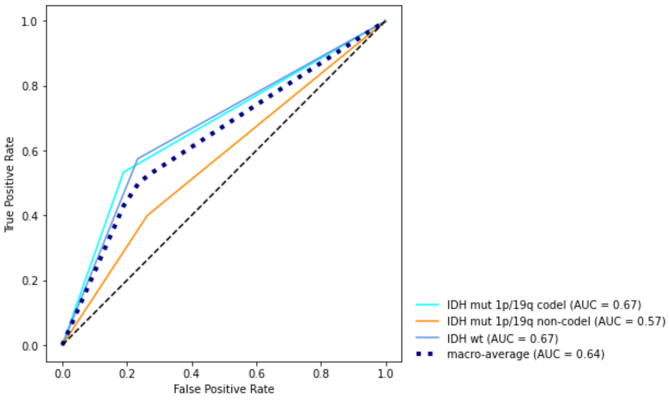

Anatomical + ADC N4

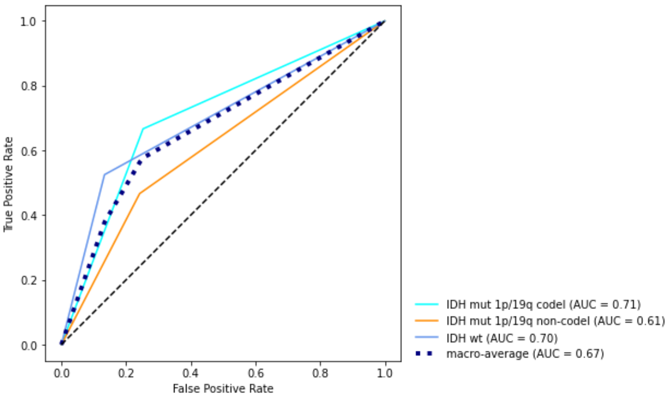

Anatomical + ADC N4/zscore

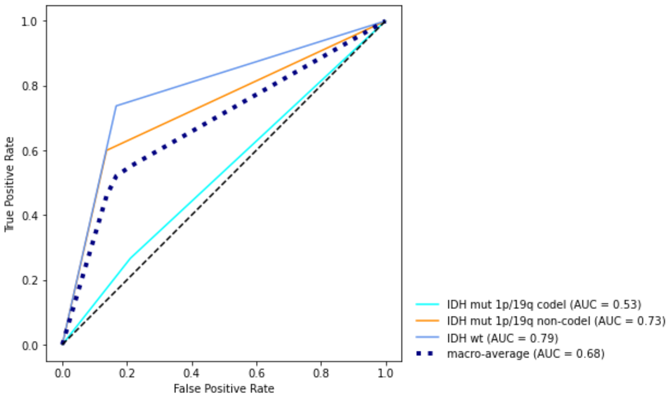

E (SVM)

Anatomical

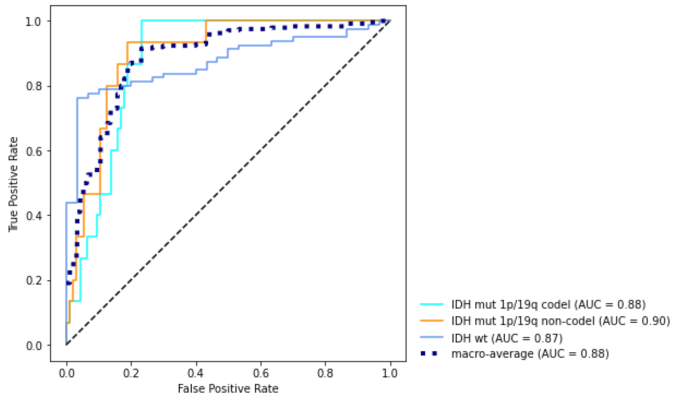

Anatomical + ADC naiv

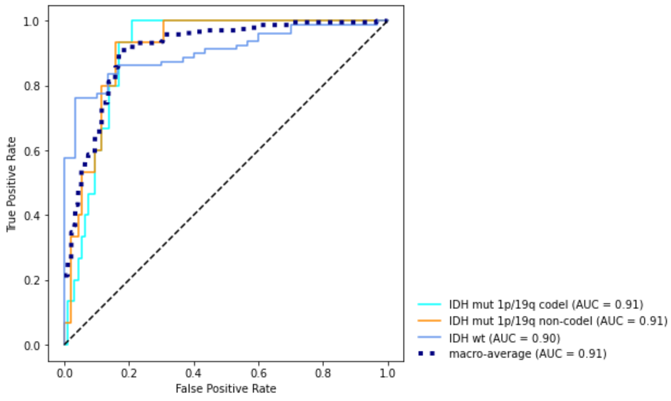

Anatomical + ADC N4

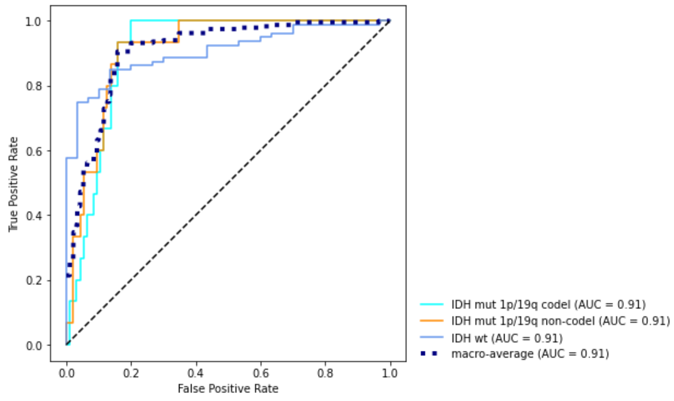

Anatomical + ADC N4/zscore

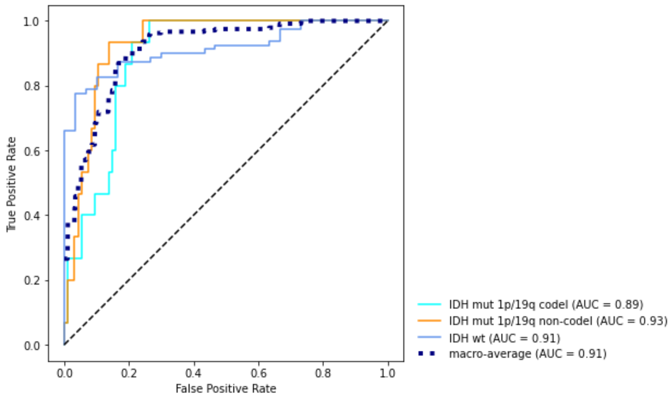

F (Random Forest)

Anatomical

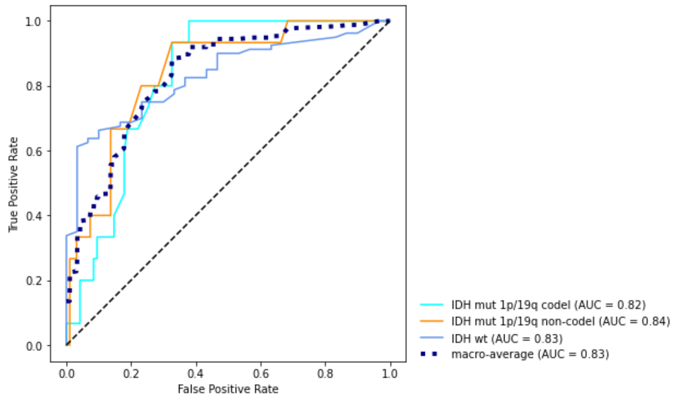

Anatomical + ADC naiv

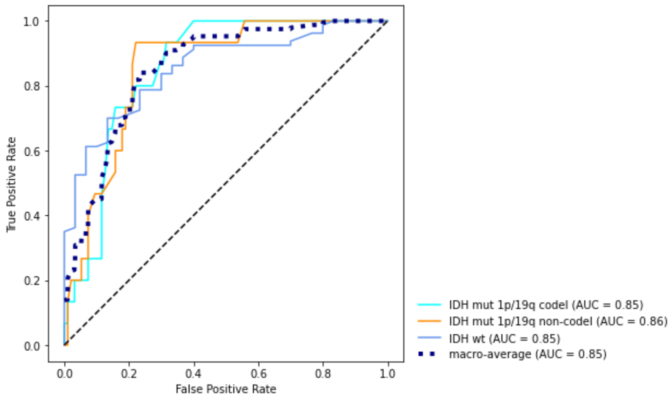

Anatomical + ADC N4

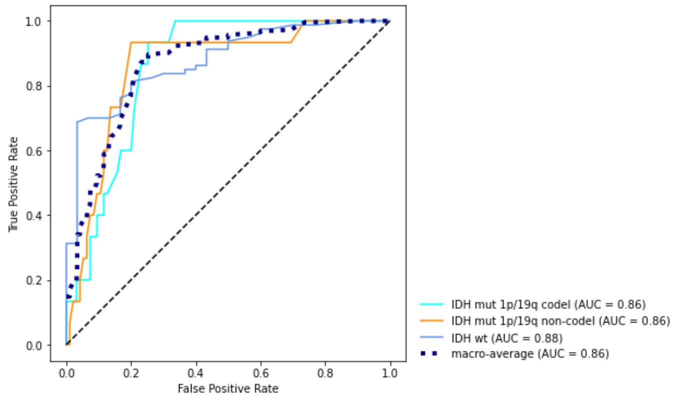

Anatomical + ADC N4/zscore

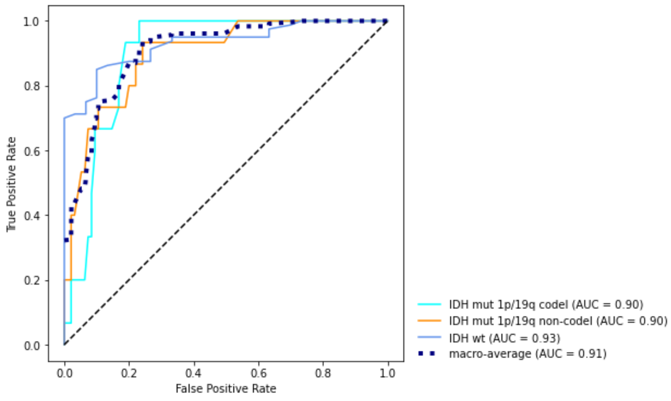

G (Extra-trees classifier)

Anatomical

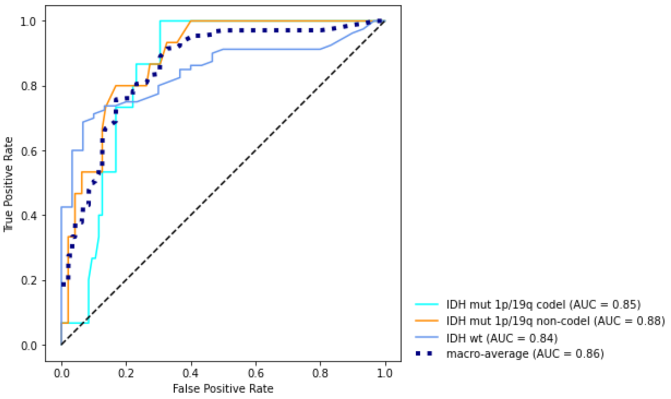

Anatomical + ADC naiv

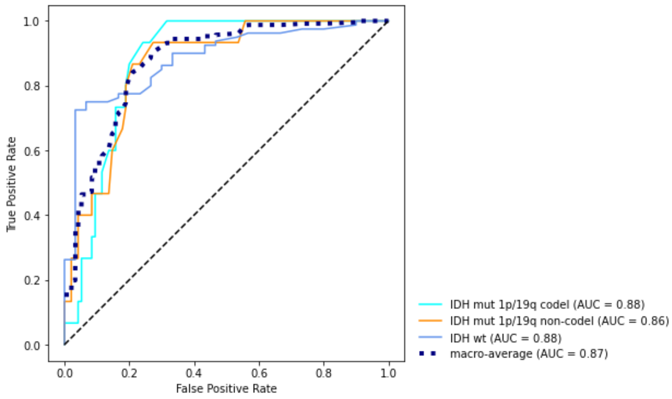

Anatomical + ADC N4

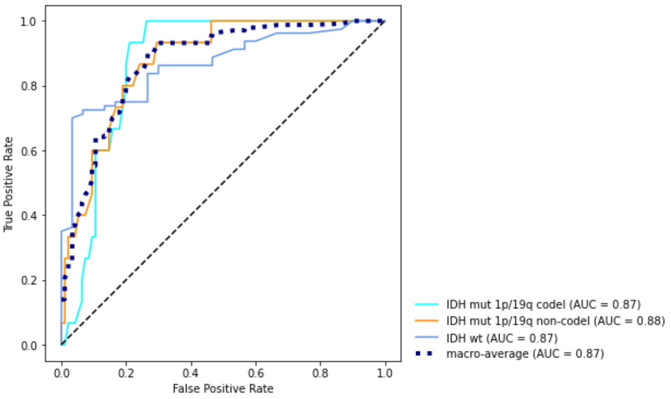

Anatomical + ADC N4/zscore

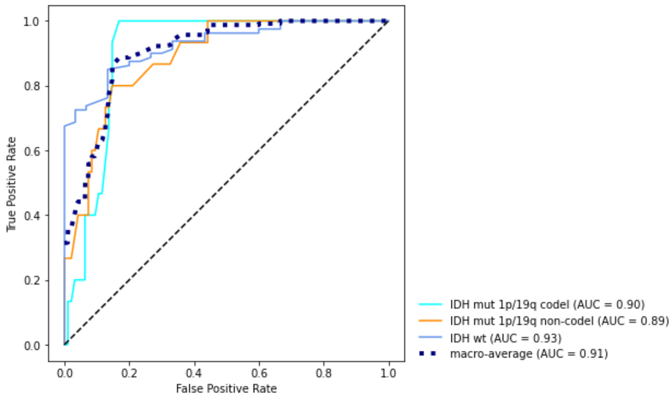

H (eXtreme Gradient)

Anatomical

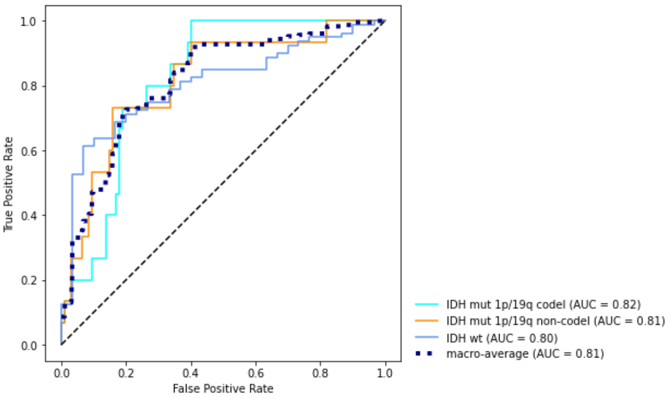

Anatomical + ADC naiv

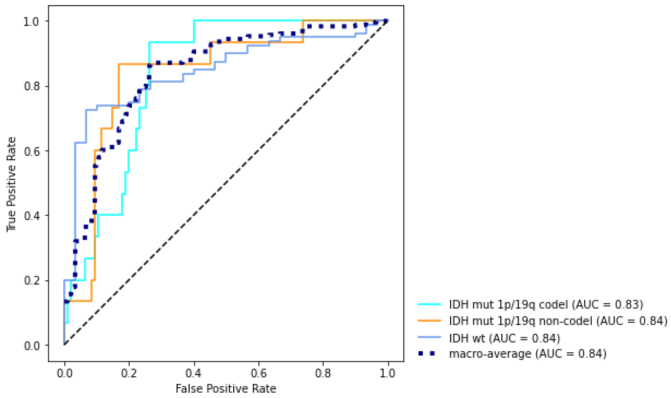

Anatomical + ADC N4

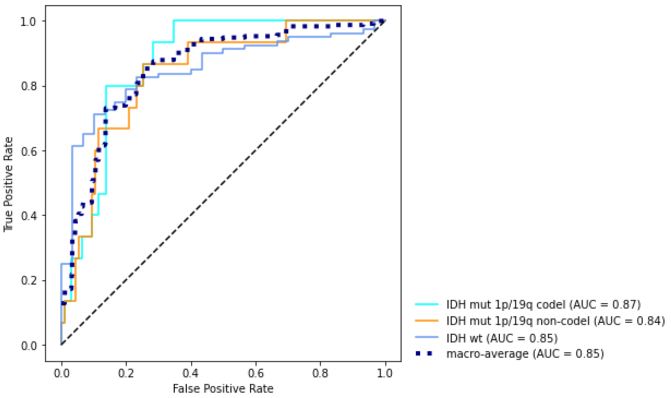

Anatomical + ADC N4/zscore

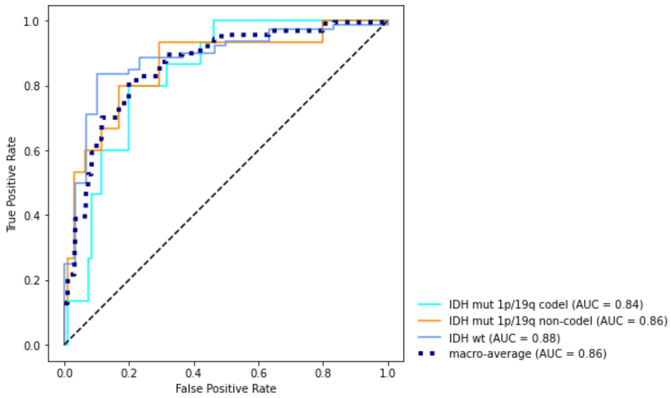

Supplementary Figure 2

A (Logistic Regression)

Anatomical

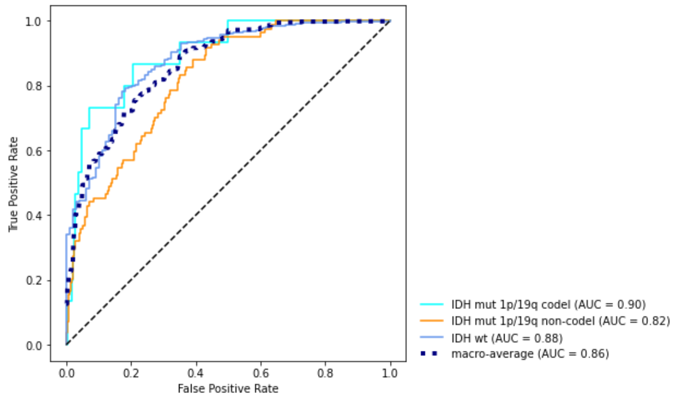

Anatomical + ADC naiv

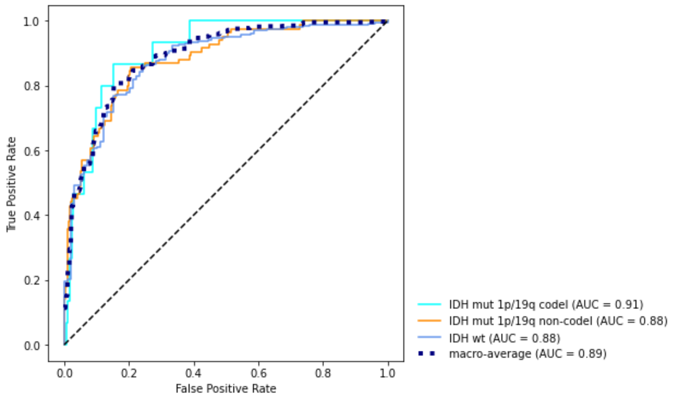

Anatomical + ADC N4

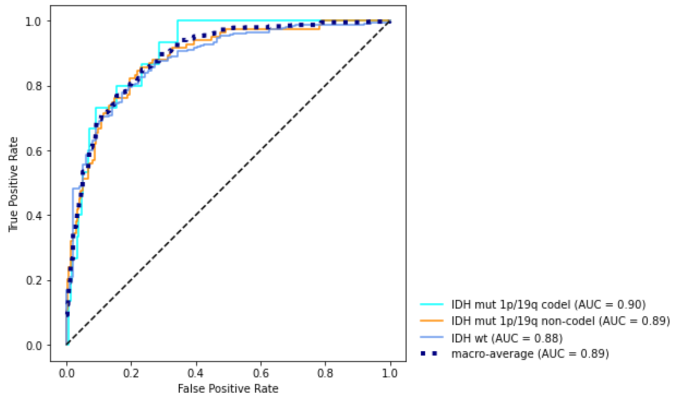

Anatomical + ADC N4/zscore

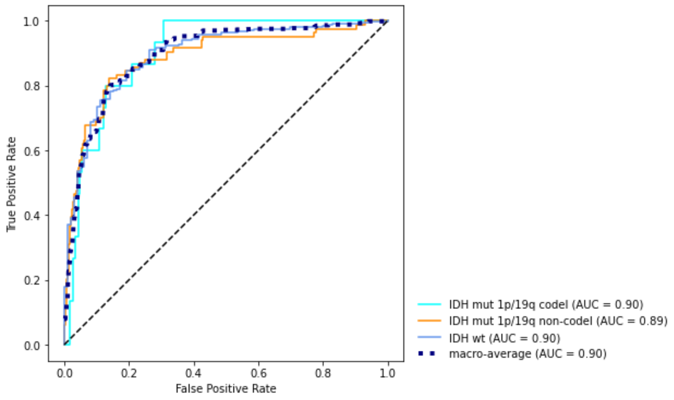

B (Linear Discriminant Analysis)

Anatomical

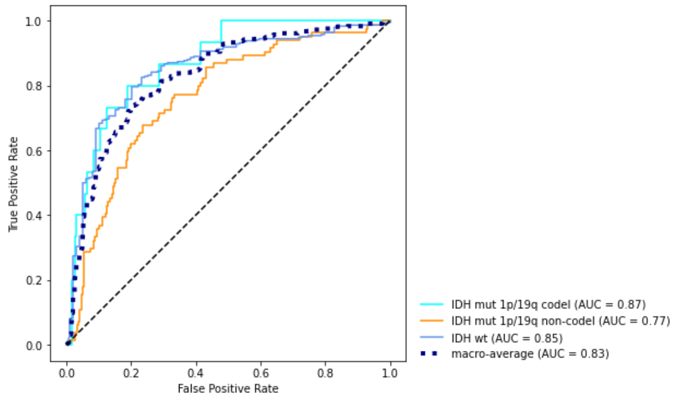

Anatomical + ADC naiv

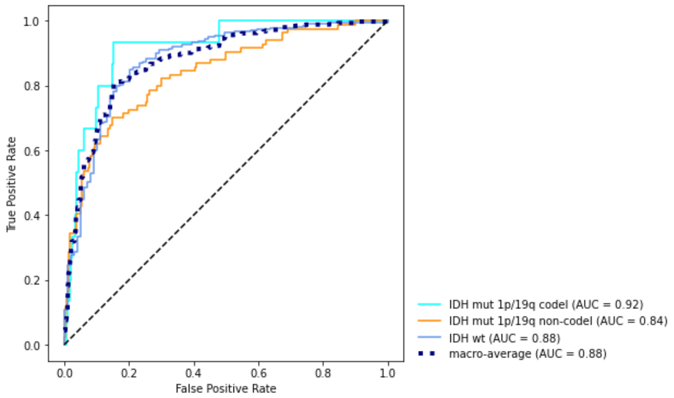

Anatomical + ADC N4

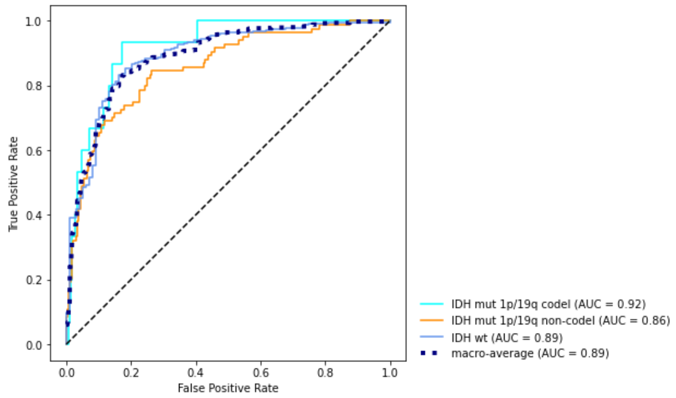

Anatomical + ADC N4/zscore

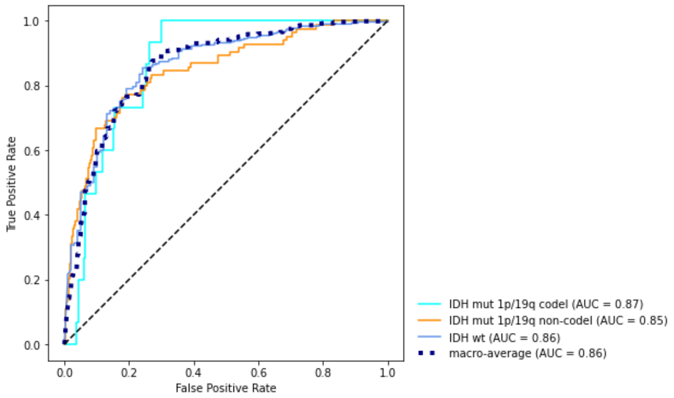

C (k-nearest neighbor)

Anatomical

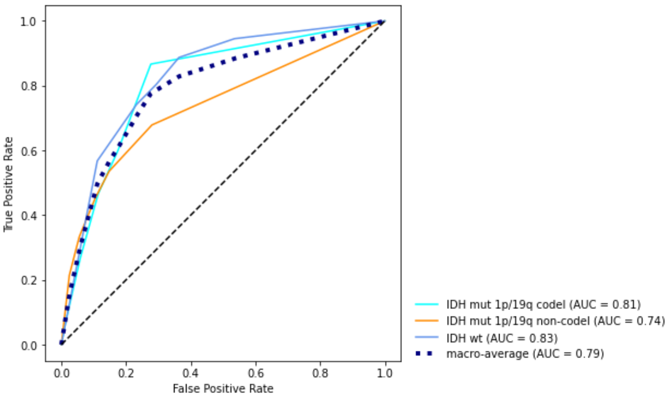

Anatomical + ADC naiv

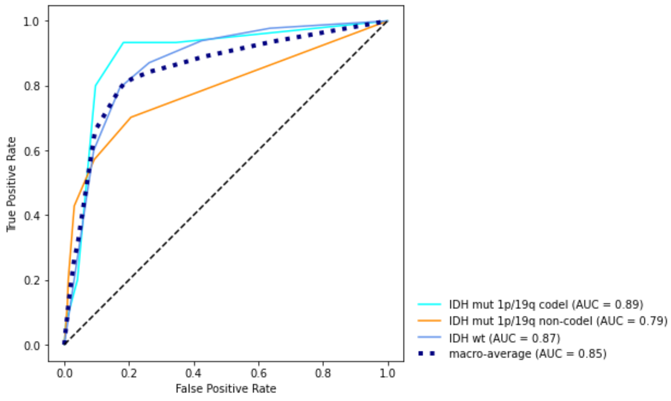

Anatomical + ADC N4

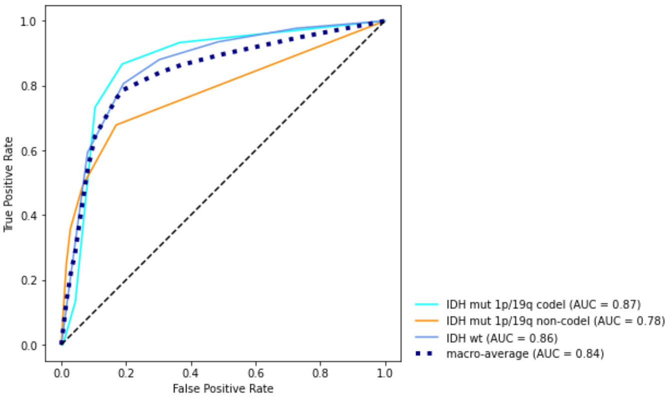

Anatomical + ADC N4/zscore

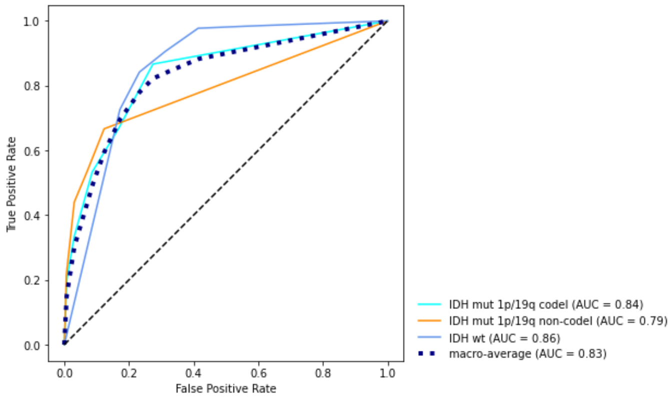

D (Decision tree)

Anatomical

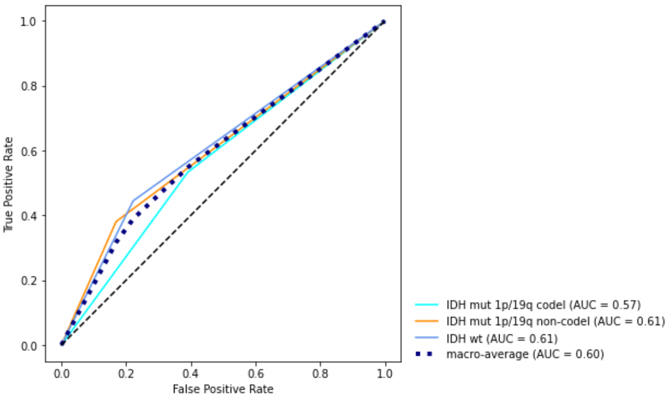

Anatomical + ADC naiv

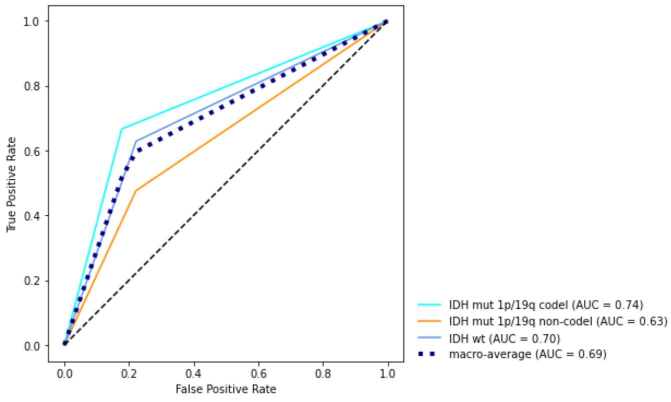

Anatomical + ADC N4

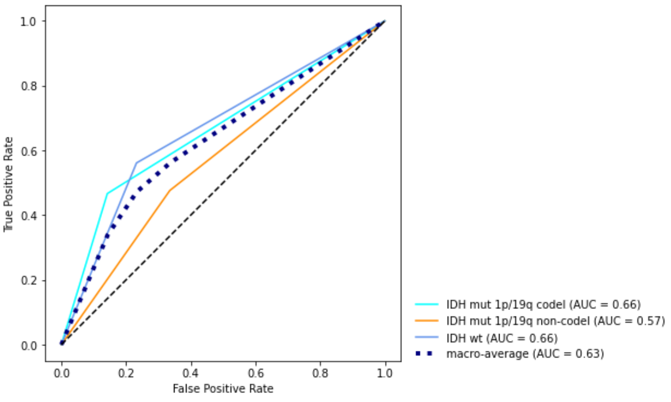

Anatomical + ADC N4/zscore

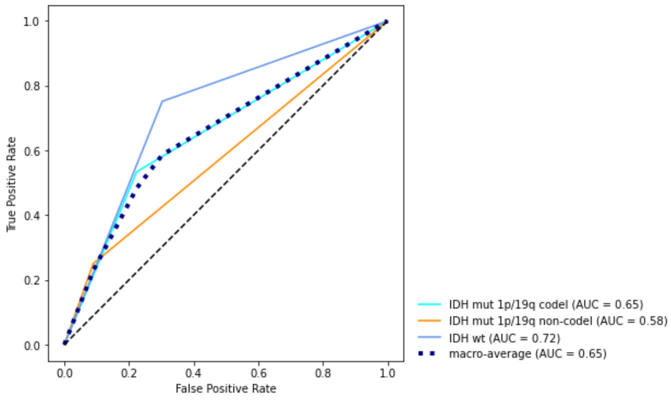

E (SVM)

Anatomical

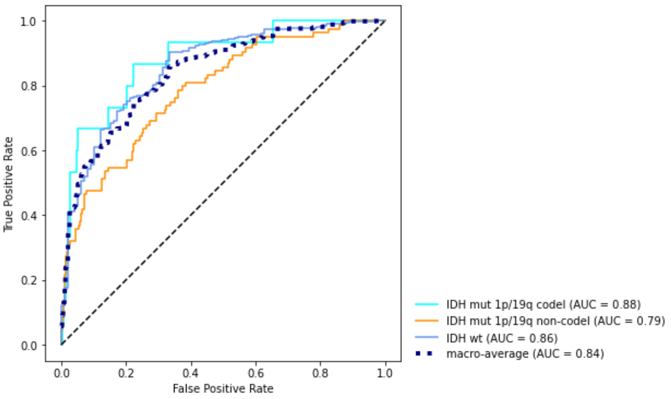

Anatomical + ADC naiv

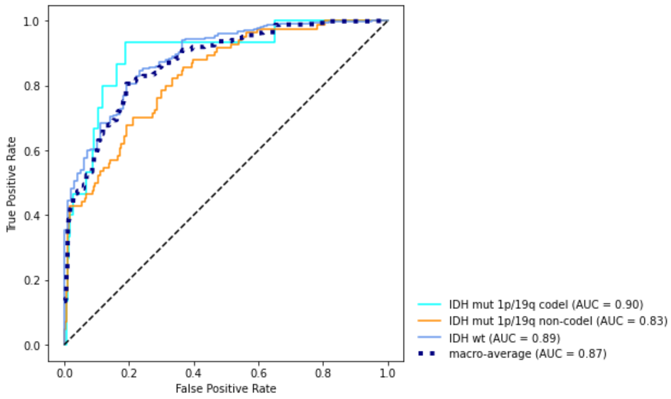

Anatomical + ADC N4

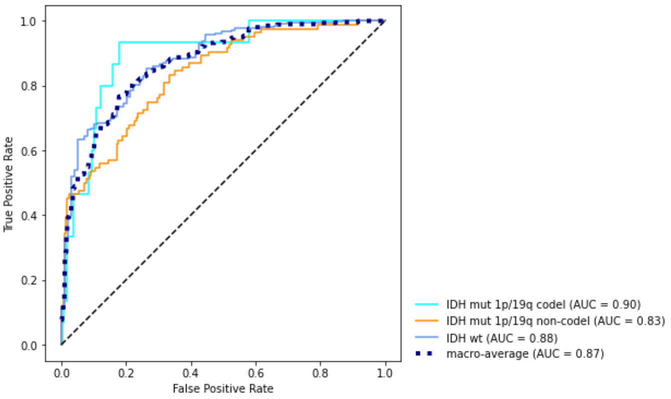

Anatomical + ADC N4/zscore

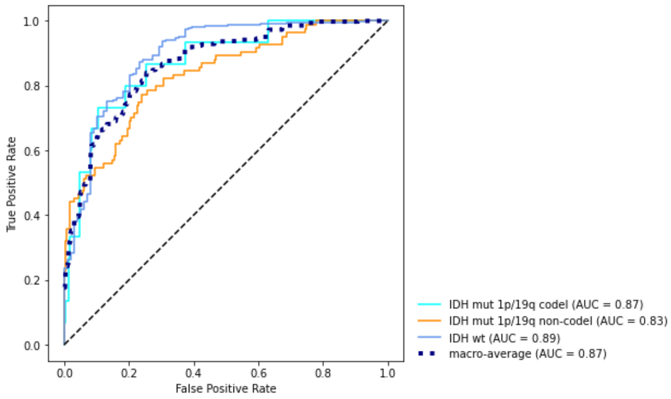

F (Random Forest)

Anatomical

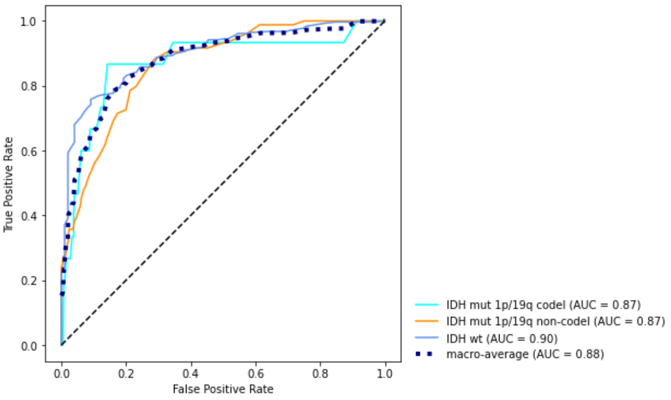

Anatomical + ADC naiv

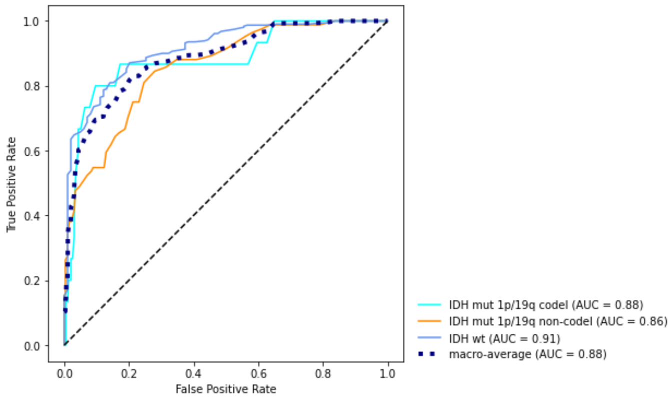

Anatomical + ADC N4

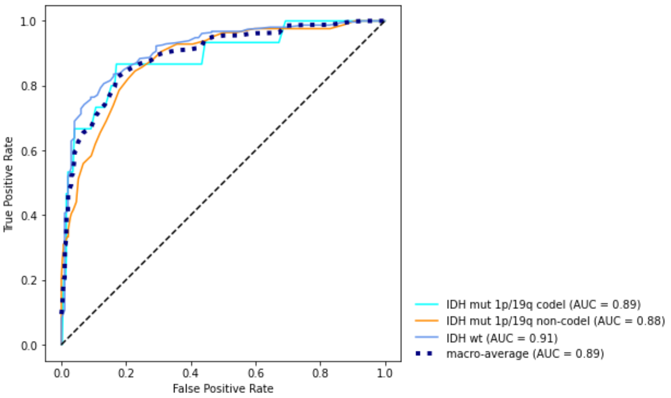

Anatomical + ADC N4/zscore

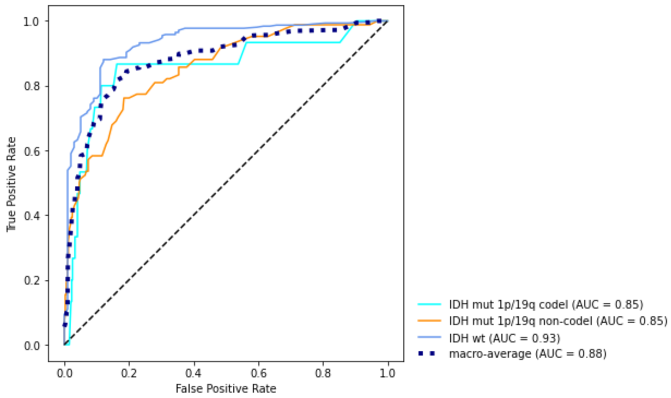

G (Extra-trees classifier)

Anatomical

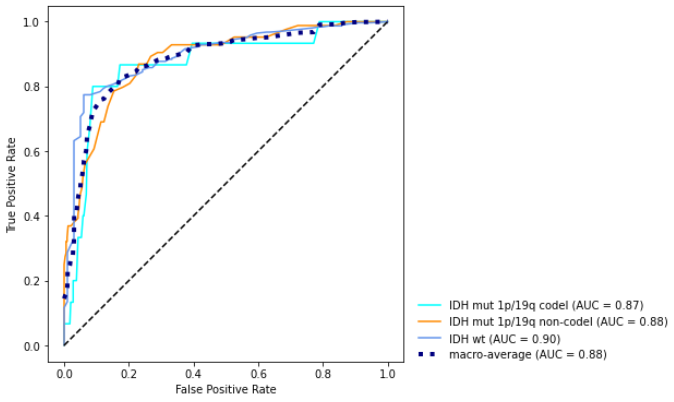

Anatomical + ADC naiv

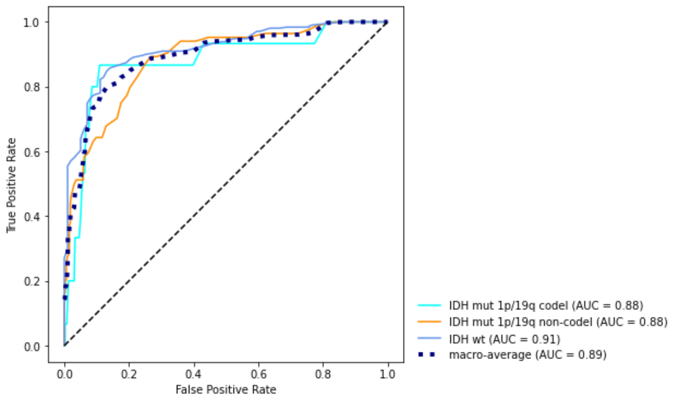

Anatomical + ADC N4

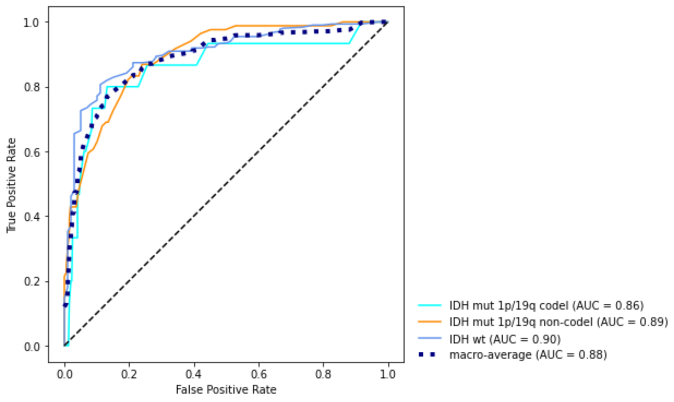

Anatomical + ADC N4/zscore

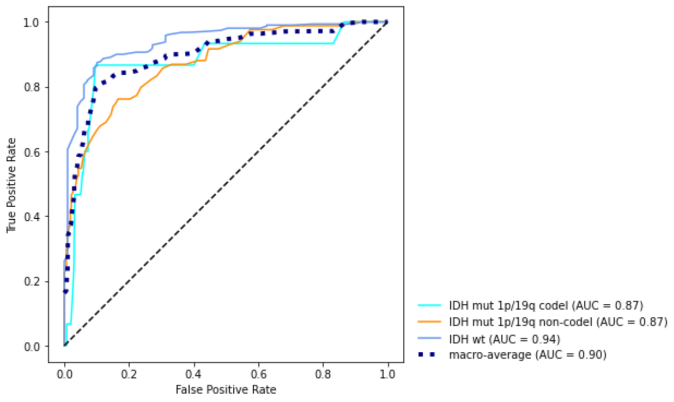

H (eXtreme Gradient)

Anatomical

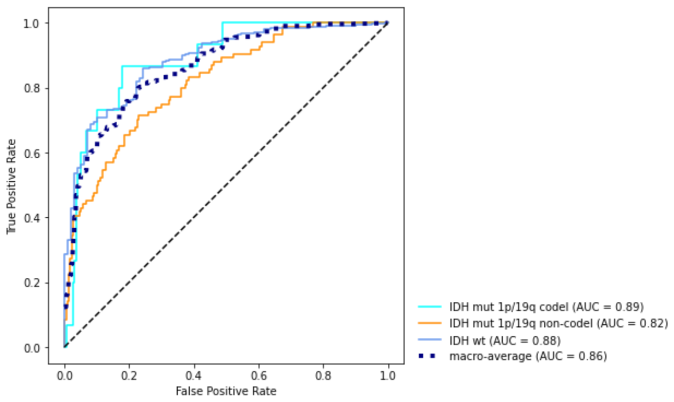

Anatomical + ADC naiv

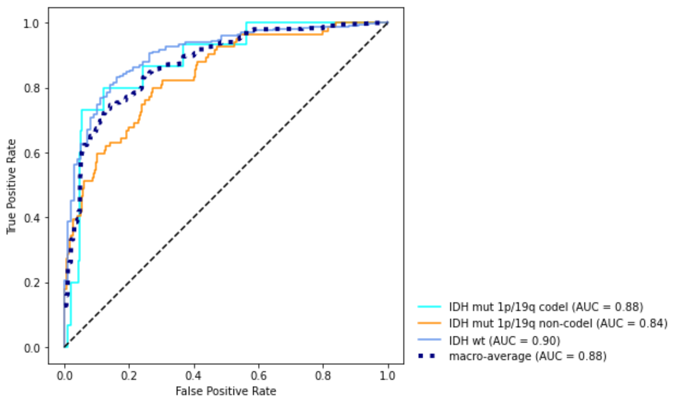

Anatomical + ADC N4

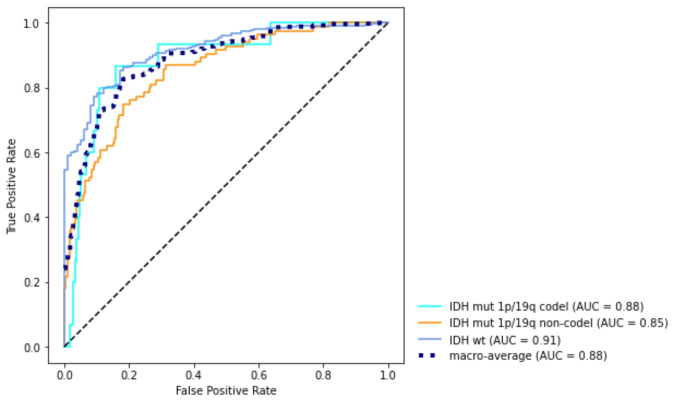

Anatomical + ADC N4/zscore

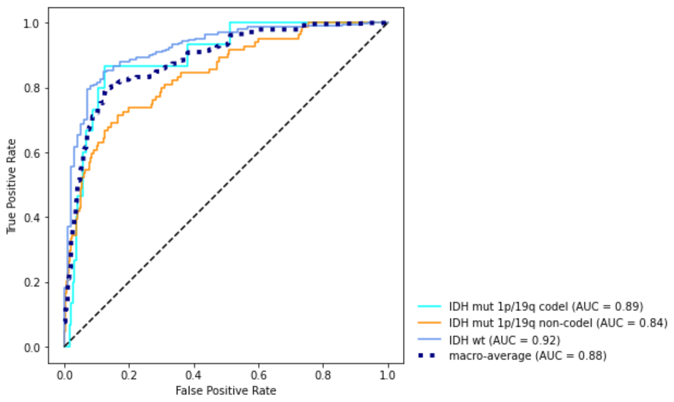

Supplement: vdae043_suppl_Supplementary_Material [file vdae043_suppl_Supplementary_Material.zip › Supplementary_Figures_NOA-D-23-00171_R1.pdf]
