# Supplementary material for: Advancing noninvasive glioma classification with diffusion radiomics: Exploring the impact of signal intensity normalization
Source: Neurooncol Adv. 2024 Mar 22;6(1):vdae043. doi: 10.1093/noajnl/vdae043 (PMC11003539; doi:10.1093/noajnl/vdae043)
Supplement: vdae043_suppl_Supplementary_Material [file vdae043_suppl_Supplementary_Material.zip › Supplementary_Materials_and_Methods.docx]

**Data Supplement**

**Supplementary Materials and Methods**

*MR acquisition protocol Heidelberg University Hospital*

Sequence parameters for T1 and cT1 MP-RAGE (3D sagittal or axial) were as follows: TI = 900–1100 ms, TE = 3–4 ms, TR = 1710–2250 ms and FA = 15°; for T2 (2D, axial): TE = 85–88 ms; TR = 2740–5950 ms; section thickness, 5 mm; spacing, 5.5mm; for FLAIR (2D, axial): TI = 2400–2500 ms; TE = 85–135 ms; TR = 8500–10 000 ms; section thickness, 5 mm; spacing, 5.5 mm.

*MR acquisition protocol UCSF*

The brain tumor MRI protocol utilized a 3.0-Tesla Discovery 750 scanner from GE Healthcare, equipped with an eight-channel head coil by Invivo. This protocol encompassed various imaging techniques: T2-weighted imaging, T2-weighted FLAIR (Fluid-Attenuated Inversion Recovery) imaging, susceptibility-weighted imaging, diffusion-weighted imaging, pre- and post-contrast T1-weighted imaging, arterial spin labeling, and 55-direction high angular resolution diffusion imaging. DWI: axial spin echo (10000/99; section thickness, 2 mm; matrix, 256 × 256; FOV, 23 cm; NSA, 1; b value =1000 sec/mm 2; 3 directions).
