# Supplementary material for: Advancing noninvasive glioma classification with diffusion radiomics: Exploring the impact of signal intensity normalization
Source: Neurooncol Adv. 2024 Mar 22;6(1):vdae043. doi: 10.1093/noajnl/vdae043 (PMC11003539; doi:10.1093/noajnl/vdae043)
Supplement: vdae043_suppl_Supplementary_Material [file vdae043_suppl_Supplementary_Material.zip › Supplementary_Table_7.docx]

**HD – LR**

| Without ADC | | **Predicted class** | | |
| --- | --- | --- | --- | --- |
|  |  | **IDH mut**  **1p19q codel** | **IDH mut**  **1p19q non-codel** | **IDH wt** |
| **Actual class** | **IDH mut**  **1p19q codel** | 11 | 3 | 1 |
|  |  |  |  |  |
|  | **IDH mut**  **1p19q non-codel** | 3 | 10 | 2 |
|  |  |  |  |  |
|  | **IDH wt** | 13 | 9 | 58 |
|  |  |  |  |  |

| + ADC naiv | | **Predicted class** | | |
| --- | --- | --- | --- | --- |
|  |  | **IDH mut**  **1p19q codel** | **IDH mut**  **1p19q non-codel** | **IDH wt** |
| **Actual class** | **IDH mut**  **1p19q codel** | 9 | 6 | 0 |
|  |  |  |  |  |
|  | **IDH mut**  **1p19q non-codel** | 3 | 8 | 4 |
|  |  |  |  |  |
|  | **IDH wt** | 15 | 6 | 59 |
|  |  |  |  |  |

| + ADC N4 | | **Predicted class** | | |
| --- | --- | --- | --- | --- |
|  |  | **IDH mut**  **1p19q codel** | **IDH mut**  **1p19q non-codel** | **IDH wt** |
| **Actual class** | **IDH mut**  **1p19q codel** | 9 | 6 | 0 |
|  |  |  |  |  |
|  | **IDH mut**  **1p19q non-codel** | 3 | 9 | 3 |
|  |  |  |  |  |
|  | **IDH wt** | 15 | 6 | 59 |
|  |  |  |  |  |

| + ADC N4/Zscore | | **Predicted class** | | |
| --- | --- | --- | --- | --- |
|  |  | **IDH mut**  **1p19q codel** | **IDH mut**  **1p19q non-codel** | **IDH wt** |
| **Actual class** | **IDH mut**  **1p19q codel** | 10 | 4 | 1 |
|  |  |  |  |  |
|  | **IDH mut**  **1p19q non-codel** | 4 | 9 | 2 |
|  |  |  |  |  |
|  | **IDH wt** | 14 | 3 | 63 |
|  |  |  |  |  |

**HD – LDA**

| Without ADC | | **Predicted class** | | |
| --- | --- | --- | --- | --- |
|  |  | **IDH mut**  **1p19q codel** | **IDH mut**  **1p19q non-codel** | **IDH wt** |
| **Actual class** | **IDH mut**  **1p19q codel** | 7 | 6 | 2 |
|  |  |  |  |  |
|  | **IDH mut**  **1p19q non-codel** | 5 | 9 | 1 |
|  |  |  |  |  |
|  | **IDH wt** | 12 | 12 | 56 |
|  |  |  |  |  |

| + ADC naiv | | **Predicted class** | | |
| --- | --- | --- | --- | --- |
|  |  | **IDH mut**  **1p19q codel** | **IDH mut**  **1p19q non-codel** | **IDH wt** |
| **Actual class** | **IDH mut**  **1p19q codel** | 9 | 5 | 1 |
|  |  |  |  |  |
|  | **IDH mut**  **1p19q non-codel** | 4 | 9 | 2 |
|  |  |  |  |  |
|  | **IDH wt** | 11 | 10 | 59 |
|  |  |  |  |  |

| + ADC N4 | | **Predicted class** | | |
| --- | --- | --- | --- | --- |
|  |  | **IDH mut**  **1p19q codel** | **IDH mut**  **1p19q non-codel** | **IDH wt** |
| **Actual class** | **IDH mut**  **1p19q codel** | 9 | 5 | 1 |
|  |  |  |  |  |
|  | **IDH mut**  **1p19q non-codel** | 5 | 8 | 2 |
|  |  |  |  |  |
|  | **IDH wt** | 10 | 8 | 62 |
|  |  |  |  |  |

| + ADC N4/Zscore | | **Predicted class** | | |
| --- | --- | --- | --- | --- |
|  |  | **IDH mut**  **1p19q codel** | **IDH mut**  **1p19q non-codel** | **IDH wt** |
| **Actual class** | **IDH mut**  **1p19q codel** | 10 | 3 | 2 |
|  |  |  |  |  |
|  | **IDH mut**  **1p19q non-codel** | 4 | 9 | 2 |
|  |  |  |  |  |
|  | **IDH wt** | 14 | 4 | 62 |
|  |  |  |  |  |

**HD – KNN**

| Without ADC | | **Predicted class** | | |
| --- | --- | --- | --- | --- |
|  |  | **IDH mut**  **1p19q codel** | **IDH mut**  **1p19q non-codel** | **IDH wt** |
| **Actual class** | **IDH mut**  **1p19q codel** | 8 | 5 | 2 |
|  |  |  |  |  |
|  | **IDH mut**  **1p19q non-codel** | 3 | 10 | 2 |
|  |  |  |  |  |
|  | **IDH wt** | 14 | 10 | 56 |
|  |  |  |  |  |

| + ADC naiv | | **Predicted class** | | |
| --- | --- | --- | --- | --- |
|  |  | **IDH mut**  **1p19q codel** | **IDH mut**  **1p19q non-codel** | **IDH wt** |
| **Actual class** | **IDH mut**  **1p19q codel** | 12 | 3 | 0 |
|  |  |  |  |  |
|  | **IDH mut**  **1p19q non-codel** | 4 | 10 | 1 |
|  |  |  |  |  |
|  | **IDH wt** | 11 | 9 | 60 |
|  |  |  |  |  |

| + ADC N4 | | **Predicted class** | | |
| --- | --- | --- | --- | --- |
|  |  | **IDH mut**  **1p19q codel** | **IDH mut**  **1p19q non-codel** | **IDH wt** |
| **Actual class** | **IDH mut**  **1p19q codel** | 11 | 3 | 1 |
|  |  |  |  |  |
|  | **IDH mut**  **1p19q non-codel** | 4 | 10 | 1 |
|  |  |  |  |  |
|  | **IDH wt** | 11 | 7 | 62 |
|  |  |  |  |  |

| + ADC N4/Zscore | | **Predicted class** | | |
| --- | --- | --- | --- | --- |
|  |  | **IDH mut**  **1p19q codel** | **IDH mut**  **1p19q non-codel** | **IDH wt** |
| **Actual class** | **IDH mut**  **1p19q codel** | 10 | 4 | 1 |
|  |  |  |  |  |
|  | **IDH mut**  **1p19q non-codel** | 4 | 10 | 1 |
|  |  |  |  |  |
|  | **IDH wt** | 13 | 4 | 63 |
|  |  |  |  |  |

**HD – CART**

| Without ADC | | **Predicted class** | | |
| --- | --- | --- | --- | --- |
|  |  | **IDH mut**  **1p19q codel** | **IDH mut**  **1p19q non-codel** | **IDH wt** |
| **Actual class** | **IDH mut**  **1p19q codel** | 9 | 4 | 2 |
|  |  |  |  |  |
|  | **IDH mut**  **1p19q non-codel** | 7 | 3 | 5 |
|  |  |  |  |  |
|  | **IDH wt** | 19 | 11 | 50 |
|  |  |  |  |  |

| + ADC naiv | | **Predicted class** | | |
| --- | --- | --- | --- | --- |
|  |  | **IDH mut**  **1p19q codel** | **IDH mut**  **1p19q non-codel** | **IDH wt** |
| **Actual class** | **IDH mut**  **1p19q codel** | 8 | 5 | 2 |
|  |  |  |  |  |
|  | **IDH mut**  **1p19q non-codel** | 4 | 6 | 5 |
|  |  |  |  |  |
|  | **IDH wt** | 14 | 20 | 46 |
|  |  |  |  |  |

| + ADC N4 | | **Predicted class** | | |
| --- | --- | --- | --- | --- |
|  |  | **IDH mut**  **1p19q codel** | **IDH mut**  **1p19q non-codel** | **IDH wt** |
| **Actual class** | **IDH mut**  **1p19q codel** | 10 | 4 | 1 |
|  |  |  |  |  |
|  | **IDH mut**  **1p19q non-codel** | 5 | 7 | 3 |
|  |  |  |  |  |
|  | **IDH wt** | 19 | 19 | 42 |
|  |  |  |  |  |

| ADC N4/Zscore | | **Predicted class** | | |
| --- | --- | --- | --- | --- |
|  |  | **IDH mut**  **1p19q codel** | **IDH mut**  **1p19q non-codel** | **IDH wt** |
| **Actual class** | **IDH mut**  **1p19q codel** | 4 | 7 | 4 |
|  |  |  |  |  |
|  | **IDH mut**  **1p19q non-codel** | 5 | 9 | 1 |
|  |  |  |  |  |
|  | **IDH wt** | 15 | 6 | 59 |
|  |  |  |  |  |

**HD – NB**

| Without ADC | | **Predicted class** | | |
| --- | --- | --- | --- | --- |
|  |  | **IDH mut**  **1p19q codel** | **IDH mut**  **1p19q non-codel** | **IDH wt** |
| **Actual class** | **IDH mut**  **1p19q codel** | 11 | 4 | 0 |
|  |  |  |  |  |
|  | **IDH mut**  **1p19q non-codel** | 3 | 9 | 3 |
|  |  |  |  |  |
|  | **IDH wt** | 12 | 13 | 55 |
|  |  |  |  |  |

| + ADC naiv | | **Predicted class** | | |
| --- | --- | --- | --- | --- |
|  |  | **IDH mut**  **1p19q codel** | **IDH mut**  **1p19q non-codel** | **IDH wt** |
| **Actual class** | **IDH mut**  **1p19q codel** | 10 | 5 | 0 |
|  |  |  |  |  |
|  | **IDH mut**  **1p19q non-codel** | 4 | 9 | 2 |
|  |  |  |  |  |
|  | **IDH wt** | 12 | 13 | 55 |
|  |  |  |  |  |

| + ADC N4 | | **Predicted class** | | |
| --- | --- | --- | --- | --- |
|  |  | **IDH mut**  **1p19q codel** | **IDH mut**  **1p19q non-codel** | **IDH wt** |
| **Actual class** | **IDH mut**  **1p19q codel** | 10 | 5 | 0 |
|  |  |  |  |  |
|  | **IDH mut**  **1p19q non-codel** | 4 | 9 | 2 |
|  |  |  |  |  |
|  | **IDH wt** | 12 | 13 | 55 |
|  |  |  |  |  |

| + ADC N4/Zscore | | **Predicted class** | | |
| --- | --- | --- | --- | --- |
|  |  | **IDH mut**  **1p19q codel** | **IDH mut**  **1p19q non-codel** | **IDH wt** |
| **Actual class** | **IDH mut**  **1p19q codel** | 11 | 4 | 0 |
|  |  |  |  |  |
|  | **IDH mut**  **1p19q non-codel** | 3 | 10 | 2 |
|  |  |  |  |  |
|  | **IDH wt** | 15 | 6 | 59 |
|  |  |  |  |  |

**HD – SVM**

| Without ADC | | **Predicted class** | | |
| --- | --- | --- | --- | --- |
|  |  | **IDH mut**  **1p19q codel** | **IDH mut**  **1p19q non-codel** | **IDH wt** |
| **Actual class** | **IDH mut**  **1p19q codel** | 9 | 5 | 1 |
|  |  |  |  |  |
|  | **IDH mut**  **1p19q non-codel** | 4 | 7 | 4 |
|  |  |  |  |  |
|  | **IDH wt** | 10 | 7 | 63 |
|  |  |  |  |  |

| + ADC naiv | | **Predicted class** | | |
| --- | --- | --- | --- | --- |
|  |  | **IDH mut**  **1p19q codel** | **IDH mut**  **1p19q non-codel** | **IDH wt** |
| **Actual class** | **IDH mut**  **1p19q codel** | 10 | 4 | 1 |
|  |  |  |  |  |
|  | **IDH mut**  **1p19q non-codel** | 4 | 7 | 4 |
|  |  |  |  |  |
|  | **IDH wt** | 7 | 5 | 68 |
|  |  |  |  |  |

| + ADC N4 | | **Predicted class** | | |
| --- | --- | --- | --- | --- |
|  |  | **IDH mut**  **1p19q codel** | **IDH mut**  **1p19q non-codel** | **IDH wt** |
| **Actual class** | **IDH mut**  **1p19q codel** | 11 | 4 | 0 |
|  |  |  |  |  |
|  | **IDH mut**  **1p19q non-codel** | 4 | 7 | 4 |
|  |  |  |  |  |
|  | **IDH wt** | 7 | 5 | 68 |
|  |  |  |  |  |

| + ADC N4/Zscore | | **Predicted class** | | |
| --- | --- | --- | --- | --- |
|  |  | **IDH mut**  **1p19q codel** | **IDH mut**  **1p19q non-codel** | **IDH wt** |
| **Actual class** | **IDH mut**  **1p19q codel** | 10 | 5 | 0 |
|  |  |  |  |  |
|  | **IDH mut**  **1p19q non-codel** | 3 | 9 | 3 |
|  |  |  |  |  |
|  | **IDH wt** | 12 | 3 | 65 |
|  |  |  |  |  |

**HD – RF**

| Without ADC | | **Predicted class** | | |
| --- | --- | --- | --- | --- |
|  |  | **IDH mut**  **1p19q codel** | **IDH mut**  **1p19q non-codel** | **IDH wt** |
| **Actual class** | **IDH mut**  **1p19q codel** | 7 | 5 | 3 |
|  |  |  |  |  |
|  | **IDH mut**  **1p19q non-codel** | 4 | 7 | 4 |
|  |  |  |  |  |
|  | **IDH wt** | 15 | 5 | 60 |
|  |  |  |  |  |

| + ADC naiv | | **Predicted class** | | |
| --- | --- | --- | --- | --- |
|  |  | **IDH mut**  **1p19q codel** | **IDH mut**  **1p19q non-codel** | **IDH wt** |
| **Actual class** | **IDH mut**  **1p19q codel** | 8 | 4 | 3 |
|  |  |  |  |  |
|  | **IDH mut**  **1p19q non-codel** | 2 | 7 | 6 |
|  |  |  |  |  |
|  | **IDH wt** | 11 | 4 | 65 |
|  |  |  |  |  |

| + ADC N4 | | **Predicted class** | | |
| --- | --- | --- | --- | --- |
|  |  | **IDH mut**  **1p19q codel** | **IDH mut**  **1p19q non-codel** | **IDH wt** |
| **Actual class** | **IDH mut**  **1p19q codel** | 8 | 4 | 3 |
|  |  |  |  |  |
|  | **IDH mut**  **1p19q non-codel** | 3 | 9 | 3 |
|  |  |  |  |  |
|  | **IDH wt** | 13 | 4 | 63 |
|  |  |  |  |  |

| + ADC N4/Zscore | | **Predicted class** | | |
| --- | --- | --- | --- | --- |
|  |  | **IDH mut**  **1p19q codel** | **IDH mut**  **1p19q non-codel** | **IDH wt** |
| **Actual class** | **IDH mut**  **1p19q codel** | 12 | 3 | 0 |
|  |  |  |  |  |
|  | **IDH mut**  **1p19q non-codel** | 2 | 10 | 3 |
|  |  |  |  |  |
|  | **IDH wt** | 12 | 3 | 65 |
|  |  |  |  |  |

**HD – ET**

| Without ADC | | **Predicted class** | | |
| --- | --- | --- | --- | --- |
|  |  | **IDH mut**  **1p19q codel** | **IDH mut**  **1p19q non-codel** | **IDH wt** |
| **Actual class** | **IDH mut**  **1p19q codel** | 11 | 3 | 1 |
|  |  |  |  |  |
|  | **IDH mut**  **1p19q non-codel** | 4 | 8 | 3 |
|  |  |  |  |  |
|  | **IDH wt** | 16 | 4 | 60 |
|  |  |  |  |  |

| + ADC naiv | | **Predicted class** | | |
| --- | --- | --- | --- | --- |
|  |  | **IDH mut**  **1p19q codel** | **IDH mut**  **1p19q non-codel** | **IDH wt** |
| **Actual class** | **IDH mut**  **1p19q codel** | 9 | 4 | 2 |
|  |  |  |  |  |
|  | **IDH mut**  **1p19q non-codel** | 4 | 6 | 5 |
|  |  |  |  |  |
|  | **IDH wt** | 13 | 4 | 63 |
|  |  |  |  |  |

| + ADC N4 | | **Predicted class** | | |
| --- | --- | --- | --- | --- |
|  |  | **IDH mut**  **1p19q codel** | **IDH mut**  **1p19q non-codel** | **IDH wt** |
| **Actual class** | **IDH mut**  **1p19q codel** | 11 | 2 | 2 |
|  |  |  |  |  |
|  | **IDH mut**  **1p19q non-codel** | 3 | 6 | 6 |
|  |  |  |  |  |
|  | **IDH wt** | 13 | 5 | 62 |
|  |  |  |  |  |

| + ADC N4/Zscore | | **Predicted class** | | |
| --- | --- | --- | --- | --- |
|  |  | **IDH mut**  **1p19q codel** | **IDH mut**  **1p19q non-codel** | **IDH wt** |
| **Actual class** | **IDH mut**  **1p19q codel** | 11 | 4 | 0 |
|  |  |  |  |  |
|  | **IDH mut**  **1p19q non-codel** | 4 | 7 | 4 |
|  |  |  |  |  |
|  | **IDH wt** | 12 | 3 | 65 |
|  |  |  |  |  |

**HD – XGBC**

| Without ADC | | **Predicted class** | | |
| --- | --- | --- | --- | --- |
|  |  | **IDH mut**  **1p19q codel** | **IDH mut**  **1p19q non-codel** | **IDH wt** |
| **Actual class** | **IDH mut**  **1p19q codel** | 9 | 4 | 2 |
|  |  |  |  |  |
|  | **IDH mut**  **1p19q non-codel** | 2 | 8 | 5 |
|  |  |  |  |  |
|  | **IDH wt** | 15 | 8 | 57 |
|  |  |  |  |  |

| + ADC naiv | | **Predicted class** | | |
| --- | --- | --- | --- | --- |
|  |  | **IDH mut**  **1p19q codel** | **IDH mut**  **1p19q non-codel** | **IDH wt** |
| **Actual class** | **IDH mut**  **1p19q codel** | 6 | 6 | 3 |
|  |  |  |  |  |
|  | **IDH mut**  **1p19q non-codel** | 3 | 6 | 6 |
|  |  |  |  |  |
|  | **IDH wt** | 12 | 3 | 65 |
|  |  |  |  |  |

| + ADC N4 | | **Predicted class** | | |
| --- | --- | --- | --- | --- |
|  |  | **IDH mut**  **1p19q codel** | **IDH mut**  **1p19q non-codel** | **IDH wt** |
| **Actual class** | **IDH mut**  **1p19q codel** | 12 | 2 | 1 |
|  |  |  |  |  |
|  | **IDH mut**  **1p19q non-codel** | 3 | 5 | 7 |
|  |  |  |  |  |
|  | **IDH wt** | 11 | 3 | 66 |
|  |  |  |  |  |

| + ADC N4/Zscore | | **Predicted class** | | |
| --- | --- | --- | --- | --- |
|  |  | **IDH mut**  **1p19q codel** | **IDH mut**  **1p19q non-codel** | **IDH wt** |
| **Actual class** | **IDH mut**  **1p19q codel** | 9 | 5 | 1 |
|  |  |  |  |  |
|  | **IDH mut**  **1p19q non-codel** | 4 | 9 | 2 |
|  |  |  |  |  |
|  | **IDH wt** | 12 | 3 | 65 |
|  |  |  |  |  |

**UCSF – LR**

| Without ADC | | **Predicted class** | | |
| --- | --- | --- | --- | --- |
|  |  | **IDH mut**  **1p19q codel** | **IDH mut**  **1p19q non-codel** | **IDH wt** |
| **Actual class** | **IDH mut**  **1p19q codel** | 13 | 1 | 1 |
|  |  |  |  |  |
|  | **IDH mut**  **1p19q non-codel** | 35 | 34 | 15 |
|  |  |  |  |  |
|  | **IDH wt** | 55 | 20 | 235 |
|  |  |  |  |  |

| + ADC naiv | | **Predicted class** | | |
| --- | --- | --- | --- | --- |
|  |  | **IDH mut**  **1p19q codel** | **IDH mut**  **1p19q non-codel** | **IDH wt** |
| **Actual class** | **IDH mut**  **1p19q codel** | 11 | 3 | 1 |
|  |  |  |  |  |
|  | **IDH mut**  **1p19q non-codel** | 16 | 42 | 26 |
|  |  |  |  |  |
|  | **IDH wt** | 27 | 13 | 270 |
|  |  |  |  |  |

| + ADC N4 | | **Predicted class** | | |
| --- | --- | --- | --- | --- |
|  |  | **IDH mut**  **1p19q codel** | **IDH mut**  **1p19q non-codel** | **IDH wt** |
| **Actual class** | **IDH mut**  **1p19q codel** | 10 | 3 | 2 |
|  |  |  |  |  |
|  | **IDH mut**  **1p19q non-codel** | 11 | 43 | 30 |
|  |  |  |  |  |
|  | **IDH wt** | 17 | 15 | 278 |
|  |  |  |  |  |

| + ADC N4/Zscore | | **Predicted class** | | |
| --- | --- | --- | --- | --- |
|  |  | **IDH mut**  **1p19q codel** | **IDH mut**  **1p19q non-codel** | **IDH wt** |
| **Actual class** | **IDH mut**  **1p19q codel** | 11 | 2 | 2 |
|  |  |  |  |  |
|  | **IDH mut**  **1p19q non-codel** | 28 | 27 | 29 |
|  |  |  |  |  |
|  | **IDH wt** | 23 | 3 | 284 |
|  |  |  |  |  |

**UCSF – LDA**

| Without ADC | | **Predicted class** | | |
| --- | --- | --- | --- | --- |
|  |  | **IDH mut**  **1p19q codel** | **IDH mut**  **1p19q non-codel** | **IDH wt** |
| **Actual class** | **IDH mut**  **1p19q codel** | 10 | 4 | 1 |
|  |  |  |  |  |
|  | **IDH mut**  **1p19q non-codel** | 27 | 32 | 25 |
|  |  |  |  |  |
|  | **IDH wt** | 17 | 32 | 261 |
|  |  |  |  |  |

| + ADC naiv | | **Predicted class** | | |
| --- | --- | --- | --- | --- |
|  |  | **IDH mut**  **1p19q codel** | **IDH mut**  **1p19q non-codel** | **IDH wt** |
| **Actual class** | **IDH mut**  **1p19q codel** | 10 | 2 | 3 |
|  |  |  |  |  |
|  | **IDH mut**  **1p19q non-codel** | 21 | 30 | 33 |
|  |  |  |  |  |
|  | **IDH wt** | 15 | 9 | 286 |
|  |  |  |  |  |

| + ADC N4 | | **Predicted class** | | |
| --- | --- | --- | --- | --- |
|  |  | **IDH mut**  **1p19q codel** | **IDH mut**  **1p19q non-codel** | **IDH wt** |
| **Actual class** | **IDH mut**  **1p19q codel** | 10 | 2 | 3 |
|  |  |  |  |  |
|  | **IDH mut**  **1p19q non-codel** | 18 | 32 | 34 |
|  |  |  |  |  |
|  | **IDH wt** | 12 | 9 | 289 |
|  |  |  |  |  |

| + ADC N4/Zscore | | **Predicted class** | | |
| --- | --- | --- | --- | --- |
|  |  | **IDH mut**  **1p19q codel** | **IDH mut**  **1p19q non-codel** | **IDH wt** |
| **Actual class** | **IDH mut**  **1p19q codel** | 8 | 2 | 5 |
|  |  |  |  |  |
|  | **IDH mut**  **1p19q non-codel** | 26 | 17 | 41 |
|  |  |  |  |  |
|  | **IDH wt** | 20 | 3 | 287 |
|  |  |  |  |  |

**UCSF – KNN**

| Without ADC | | **Predicted class** | | |
| --- | --- | --- | --- | --- |
|  |  | **IDH mut**  **1p19q codel** | **IDH mut**  **1p19q non-codel** | **IDH wt** |
| **Actual class** | **IDH mut**  **1p19q codel** | 7 | 4 | 4 |
|  |  |  |  |  |
|  | **IDH mut**  **1p19q non-codel** | 19 | 40 | 25 |
|  |  |  |  |  |
|  | **IDH wt** | 30 | 31 | 249 |
|  |  |  |  |  |

| + ADC naiv | | **Predicted class** | | |
| --- | --- | --- | --- | --- |
|  |  | **IDH mut**  **1p19q codel** | **IDH mut**  **1p19q non-codel** | **IDH wt** |
| **Actual class** | **IDH mut**  **1p19q codel** | 12 | 0 | 3 |
|  |  |  |  |  |
|  | **IDH mut**  **1p19q non-codel** | 23 | 38 | 23 |
|  |  |  |  |  |
|  | **IDH wt** | 25 | 15 | 270 |
|  |  |  |  |  |

| + ADC N4 | | **Predicted class** | | |
| --- | --- | --- | --- | --- |
|  |  | **IDH mut**  **1p19q codel** | **IDH mut**  **1p19q non-codel** | **IDH wt** |
| **Actual class** | **IDH mut**  **1p19q codel** | 12 | 0 | 3 |
|  |  |  |  |  |
|  | **IDH mut**  **1p19q non-codel** | 26 | 31 | 27 |
|  |  |  |  |  |
|  | **IDH wt** | 25 | 12 | 273 |
|  |  |  |  |  |

| + ADC N4/Zscore | | **Predicted class** | | |
| --- | --- | --- | --- | --- |
|  |  | **IDH mut**  **1p19q codel** | **IDH mut**  **1p19q non-codel** | **IDH wt** |
| **Actual class** | **IDH mut**  **1p19q codel** | 9 | 2 | 4 |
|  |  |  |  |  |
|  | **IDH mut**  **1p19q non-codel** | 19 | 38 | 27 |
|  |  |  |  |  |
|  | **IDH wt** | 19 | 10 | 281 |
|  |  |  |  |  |

**UCSF – CART**

| Without ADC | | **Predicted class** | | |
| --- | --- | --- | --- | --- |
|  |  | **IDH mut**  **1p19q codel** | **IDH mut**  **1p19q non-codel** | **IDH wt** |
| **Actual class** | **IDH mut**  **1p19q codel** | 8 | 5 | 2 |
|  |  |  |  |  |
|  | **IDH mut**  **1p19q non-codel** | 32 | 32 | 20 |
|  |  |  |  |  |
|  | **IDH wt** | 122 | 50 | 138 |
|  |  |  |  |  |

| + ADC naiv | | **Predicted class** | | |
| --- | --- | --- | --- | --- |
|  |  | **IDH mut**  **1p19q codel** | **IDH mut**  **1p19q non-codel** | **IDH wt** |
| **Actual class** | **IDH mut**  **1p19q codel** | 10 | 4 | 1 |
|  |  |  |  |  |
|  | **IDH mut**  **1p19q non-codel** | 23 | 40 | 21 |
|  |  |  |  |  |
|  | **IDH wt** | 47 | 68 | 195 |
|  |  |  |  |  |

| + ADC N4 | | **Predicted class** | | |
| --- | --- | --- | --- | --- |
|  |  | **IDH mut**  **1p19q codel** | **IDH mut**  **1p19q non-codel** | **IDH wt** |
| **Actual class** | **IDH mut**  **1p19q codel** | 7 | 3 | 5 |
|  |  |  |  |  |
|  | **IDH mut**  **1p19q non-codel** | 26 | 40 | 18 |
|  |  |  |  |  |
|  | **IDH wt** | 30 | 106 | 174 |
|  |  |  |  |  |

| + ADC N4/Zscore | | **Predicted class** | | |
| --- | --- | --- | --- | --- |
|  |  | **IDH mut**  **1p19q codel** | **IDH mut**  **1p19q non-codel** | **IDH wt** |
| **Actual class** | **IDH mut**  **1p19q codel** | 8 | 3 | 4 |
|  |  |  |  |  |
|  | **IDH mut**  **1p19q non-codel** | 37 | 21 | 26 |
|  |  |  |  |  |
|  | **IDH wt** | 51 | 26 | 233 |
|  |  |  |  |  |

**UCSF – NB**

| Without ADC | | **Predicted class** | | |
| --- | --- | --- | --- | --- |
|  |  | **IDH mut**  **1p19q codel** | **IDH mut**  **1p19q non-codel** | **IDH wt** |
| **Actual class** | **IDH mut**  **1p19q codel** | 10 | 2 | 3 |
|  |  |  |  |  |
|  | **IDH mut**  **1p19q non-codel** | 18 | 42 | 24 |
|  |  |  |  |  |
|  | **IDH wt** | 26 | 36 | 248 |
|  |  |  |  |  |

| + ADC naiv | | **Predicted class** | | |
| --- | --- | --- | --- | --- |
|  |  | **IDH mut**  **1p19q codel** | **IDH mut**  **1p19q non-codel** | **IDH wt** |
| **Actual class** | **IDH mut**  **1p19q codel** | 9 | 4 | 2 |
|  |  |  |  |  |
|  | **IDH mut**  **1p19q non-codel** | 24 | 37 | 23 |
|  |  |  |  |  |
|  | **IDH wt** | 24 | 44 | 242 |
|  |  |  |  |  |

| + ADC N4 | | **Predicted class** | | |
| --- | --- | --- | --- | --- |
|  |  | **IDH mut**  **1p19q codel** | **IDH mut**  **1p19q non-codel** | **IDH wt** |
| **Actual class** | **IDH mut**  **1p19q codel** | 9 | 3 | 3 |
|  |  |  |  |  |
|  | **IDH mut**  **1p19q non-codel** | 22 | 42 | 20 |
|  |  |  |  |  |
|  | **IDH wt** | 24 | 44 | 242 |
|  |  |  |  |  |

| + ADC N4/Zscore | | **Predicted class** | | |
| --- | --- | --- | --- | --- |
|  |  | **IDH mut**  **1p19q codel** | **IDH mut**  **1p19q non-codel** | **IDH wt** |
| **Actual class** | **IDH mut**  **1p19q codel** | 11 | 2 | 2 |
|  |  |  |  |  |
|  | **IDH mut**  **1p19q non-codel** | 19 | 40 | 25 |
|  |  |  |  |  |
|  | **IDH wt** | 16 | 18 | 276 |
|  |  |  |  |  |

**UCSF – SVM**

| Without ADC | | **Predicted class** | | |
| --- | --- | --- | --- | --- |
|  |  | **IDH mut**  **1p19q codel** | **IDH mut**  **1p19q non-codel** | **IDH wt** |
| **Actual class** | **IDH mut**  **1p19q codel** | 10 | 1 | 4 |
|  |  |  |  |  |
|  | **IDH mut**  **1p19q non-codel** | 16 | 36 | 32 |
|  |  |  |  |  |
|  | **IDH wt** | 13 | 19 | 278 |
|  |  |  |  |  |

| + ADC naiv | | **Predicted class** | | |
| --- | --- | --- | --- | --- |
|  |  | **IDH mut**  **1p19q codel** | **IDH mut**  **1p19q non-codel** | **IDH wt** |
| **Actual class** | **IDH mut**  **1p19q codel** | 7 | 1 | 7 |
|  |  |  |  |  |
|  | **IDH mut**  **1p19q non-codel** | 14 | 32 | 38 |
|  |  |  |  |  |
|  | **IDH wt** | 11 | 3 | 296 |
|  |  |  |  |  |

| + ADC N4 | | **Predicted class** | | |
| --- | --- | --- | --- | --- |
|  |  | **IDH mut**  **1p19q codel** | **IDH mut**  **1p19q non-codel** | **IDH wt** |
| **Actual class** | **IDH mut**  **1p19q codel** | 6 | 1 | 8 |
|  |  |  |  |  |
|  | **IDH mut**  **1p19q non-codel** | 13 | 28 | 43 |
|  |  |  |  |  |
|  | **IDH wt** | 7 | 2 | 301 |
|  |  |  |  |  |

| + ADC N4/Zscore | | **Predicted class** | | |
| --- | --- | --- | --- | --- |
|  |  | **IDH mut**  **1p19q codel** | **IDH mut**  **1p19q non-codel** | **IDH wt** |
| **Actual class** | **IDH mut**  **1p19q codel** | 8 | 1 | 6 |
|  |  |  |  |  |
|  | **IDH mut**  **1p19q non-codel** | 19 | 40 | 25 |
|  |  |  |  |  |
|  | **IDH wt** | 6 | 18 | 286 |
|  |  |  |  |  |

**UCSF – RF**

| Without ADC | | **Predicted class** | | |
| --- | --- | --- | --- | --- |
|  |  | **IDH mut**  **1p19q codel** | **IDH mut**  **1p19q non-codel** | **IDH wt** |
| **Actual class** | **IDH mut**  **1p19q codel** | 11 | 3 | 1 |
|  |  |  |  |  |
|  | **IDH mut**  **1p19q non-codel** | 29 | 41 | 14 |
|  |  |  |  |  |
|  | **IDH wt** | 49 | 16 | 245 |
|  |  |  |  |  |

| + ADC naiv | | **Predicted class** | | |
| --- | --- | --- | --- | --- |
|  |  | **IDH mut**  **1p19q codel** | **IDH mut**  **1p19q non-codel** | **IDH wt** |
| **Actual class** | **IDH mut**  **1p19q codel** | 13 | 1 | 1 |
|  |  |  |  |  |
|  | **IDH mut**  **1p19q non-codel** | 24 | 42 | 18 |
|  |  |  |  |  |
|  | **IDH wt** | 30 | 20 | 260 |
|  |  |  |  |  |

| + ADC N4 | | **Predicted class** | | |
| --- | --- | --- | --- | --- |
|  |  | **IDH mut**  **1p19q codel** | **IDH mut**  **1p19q non-codel** | **IDH wt** |
| **Actual class** | **IDH mut**  **1p19q codel** | 11 | 2 | 2 |
|  |  |  |  |  |
|  | **IDH mut**  **1p19q non-codel** | 24 | 42 | 18 |
|  |  |  |  |  |
|  | **IDH wt** | 30 | 18 | 262 |
|  |  |  |  |  |

| + ADC N4/Zscore | | **Predicted class** | | |
| --- | --- | --- | --- | --- |
|  |  | **IDH mut**  **1p19q codel** | **IDH mut**  **1p19q non-codel** | **IDH wt** |
| **Actual class** | **IDH mut**  **1p19q codel** | 11 | 2 | 2 |
|  |  |  |  |  |
|  | **IDH mut**  **1p19q non-codel** | 22 | 46 | 16 |
|  |  |  |  |  |
|  | **IDH wt** | 15 | 18 | 277 |
|  |  |  |  |  |

**UCSF – ET**

| Without ADC | | **Predicted class** | | |
| --- | --- | --- | --- | --- |
|  |  | **IDH mut**  **1p19q codel** | **IDH mut**  **1p19q non-codel** | **IDH wt** |
| **Actual class** | **IDH mut**  **1p19q codel** | 12 | 1 | 2 |
|  |  |  |  |  |
|  | **IDH mut**  **1p19q non-codel** | 20 | 42 | 22 |
|  |  |  |  |  |
|  | **IDH wt** | 34 | 14 | 262 |
|  |  |  |  |  |

| + ADC naiv | | **Predicted class** | | |
| --- | --- | --- | --- | --- |
|  |  | **IDH mut**  **1p19q codel** | **IDH mut**  **1p19q non-codel** | **IDH wt** |
| **Actual class** | **IDH mut**  **1p19q codel** | 12 | 2 | 1 |
|  |  |  |  |  |
|  | **IDH mut**  **1p19q non-codel** | 18 | 45 | 21 |
|  |  |  |  |  |
|  | **IDH wt** | 24 | 9 | 277 |
|  |  |  |  |  |

| + ADC N4 | | **Predicted class** | | |
| --- | --- | --- | --- | --- |
|  |  | **IDH mut**  **1p19q codel** | **IDH mut**  **1p19q non-codel** | **IDH wt** |
| **Actual class** | **IDH mut**  **1p19q codel** | 11 | 2 | 2 |
|  |  |  |  |  |
|  | **IDH mut**  **1p19q non-codel** | 20 | 44 | 20 |
|  |  |  |  |  |
|  | **IDH wt** | 31 | 9 | 270 |
|  |  |  |  |  |

| + ADC N4/Zscore | | **Predicted class** | | |
| --- | --- | --- | --- | --- |
|  |  | **IDH mut**  **1p19q codel** | **IDH mut**  **1p19q non-codel** | **IDH wt** |
| **Actual class** | **IDH mut**  **1p19q codel** | 11 | 2 | 2 |
|  |  |  |  |  |
|  | **IDH mut**  **1p19q non-codel** | 22 | 45 | 17 |
|  |  |  |  |  |
|  | **IDH wt** | 22 | 10 | 278 |
|  |  |  |  |  |

**UCSF – XGBC**

| Without ADC | | **Predicted class** | | |
| --- | --- | --- | --- | --- |
|  |  | **IDH mut**  **1p19q codel** | **IDH mut**  **1p19q non-codel** | **IDH wt** |
| **Actual class** | **IDH mut**  **1p19q codel** | 11 | 3 | 1 |
|  |  |  |  |  |
|  | **IDH mut**  **1p19q non-codel** | 22 | 47 | 15 |
|  |  |  |  |  |
|  | **IDH wt** | 45 | 38 | 227 |
|  |  |  |  |  |

| + ADC naiv | | **Predicted class** | | |
| --- | --- | --- | --- | --- |
|  |  | **IDH mut**  **1p19q codel** | **IDH mut**  **1p19q non-codel** | **IDH wt** |
| **Actual class** | **IDH mut**  **1p19q codel** | 12 | 3 | 0 |
|  |  |  |  |  |
|  | **IDH mut**  **1p19q non-codel** | 22 | 51 | 11 |
|  |  |  |  |  |
|  | **IDH wt** | 40 | 34 | 236 |
|  |  |  |  |  |

| + ADC N4 | | **Predicted class** | | |
| --- | --- | --- | --- | --- |
|  |  | **IDH mut**  **1p19q codel** | **IDH mut**  **1p19q non-codel** | **IDH wt** |
| **Actual class** | **IDH mut**  **1p19q codel** | 12 | 3 | 0 |
|  |  |  |  |  |
|  | **IDH mut**  **1p19q non-codel** | 23 | 51 | 10 |
|  |  |  |  |  |
|  | **IDH wt** | 37 | 31 | 242 |
|  |  |  |  |  |

| + ADC N4/Zscore | | **Predicted class** | | |
| --- | --- | --- | --- | --- |
|  |  | **IDH mut**  **1p19q codel** | **IDH mut**  **1p19q non-codel** | **IDH wt** |
| **Actual class** | **IDH mut**  **1p19q codel** | 11 | 2 | 2 |
|  |  |  |  |  |
|  | **IDH mut**  **1p19q non-codel** | 21 | 52 | 11 |
|  |  |  |  |  |
|  | **IDH wt** | 21 | 30 | 259 |
|  |  |  |  |  |
