# Supplementary material for: Advancing noninvasive glioma classification with diffusion radiomics: Exploring the impact of signal intensity normalization
Source: Neurooncol Adv. 2024 Mar 22;6(1):vdae043. doi: 10.1093/noajnl/vdae043 (PMC11003539; doi:10.1093/noajnl/vdae043)
Supplement: vdae043_suppl_Supplementary_Material [file vdae043_suppl_Supplementary_Material.zip › Supplementary_Table_8.docx]

**Supplementary Table 8**

| **Parameter** | **All Classes** | **IDH mut+1p/19q codeletion** | **IDH mut+1p/19q non-codeletion** | **IDH wt** |
| --- | --- | --- | --- | --- |
| Total no. of patients | 438 | 146 | 146 | 146 |
| Female[n(%)] | 177(40) | 69(47) | 54(37) | 54(37) |
| Mean age [y] | 53 ± 16 | 48 ± 15 | 47 ± 15 | 64 ± 13 |
