# Supplementary material for: Advancing noninvasive glioma classification with diffusion radiomics: Exploring the impact of signal intensity normalization
Source: Neurooncol Adv. 2024 Mar 22;6(1):vdae043. doi: 10.1093/noajnl/vdae043 (PMC11003539; doi:10.1093/noajnl/vdae043)
Supplement: vdae043_suppl_Supplementary_Material [file vdae043_suppl_Supplementary_Material.zip › Supplementary_Table_and_Figure_Legends.docx]

**Supplementary Table and Figure Legends**

**Supplementary Figure 1.** Classifier performance on holdout test dataset (n=110) of HD to classify IDH-wt vs. IDH-mut 1p19q codeleted vs. IDH-mut 1p19q non-codeleted via radiomic features across different conditions: anatomical sequences alone (anatomical), anatomical sequences + unnormalized ADC (anatomical + ADC naiv), anatomical sequences + ADC with N4 Bias Field Correction (anatomical + ADC N4) or anatomical sequences + ADC N4 Bias Field Correction followed by z-score normalization (anatomical + ADC N4/z-score) and eight different machine learning algorithm: Logistic Regression (A), Linear Discriminant Analysis (B), k-nearest neighbor (C), Decision tree (D), SVM (E), Random Forest (F), Extra-trees classifier (G) as well as eXtreme Gradient (H)

**Supplementary Figure 2.** Performance of external validation on UCSF (n=409) to classify IDH-wt vs. IDH-mut 1p19q codeleted vs. IDH-mut 1p19q non-codeleted across normalization conditions: anatomical sequences alone (anatomical), anatomical sequences + unnormalized ADC (anatomical + ADC naiv), anatomical sequences + ADC with N4 Bias Field Correction (anatomical + ADC N4) or anatomical sequences + ADC N4 Bias Field Correction followed by z-score normalization (anatomical + ADC N4/z-score) and eight different machine learning algorithm: Logistic Regression (A), Linear Discriminant Analysis (B), k-nearest neighbor (C), Decision tree (D), SVM (E), Random Forest (F), Extra-trees classifier (G) as well as eXtreme Gradient (H)

**Supplementary Table 1.** List of all radiomics divided by the corresponding feautre class.

**Supplementary Table 2.** Sensitivity, specificity and accuracy of various classifiers tested on the HD holdout test data set.

**Supplementary Table 3.** Sensitivity, specificity and accuracy of various classifiers tested on the external UCSF data set.

**Supplementary Table 4.** DeLong test-calculated and FDR-corrected p-values of the AUCs of IDH-mut 1p/19q codeleted class.

**Supplementary Table 5.** DeLong test-calculated and FDR-corrected p-values of the AUCs of IDH-mut 1p/19q non-codeleted class.

**Supplementary Table 6.** DeLong test-calculated and FDR-corrected p-values of the AUCs of IDH-wt class.

**Supplementary Table 7**. Confusion matrices of various classifiers tested on the HD holdout test data set and external UCSF data set.

**Supplementary Table 8**. Patient Demographic Characteristics of the training data set after undersampling the IDH-wt and oversampling the IDH-mut classes using SMOTE.
